# Discovery of dynamical heterogeneity in a supercooled magnetic monopole fluid

**DOI:** 10.1073/pnas.2528457123

**Published:** 2026-06-05

**Authors:** Jahnatta Dasini, Chaia Carroll, Jack Murphy, Catherine Dawson, Hiroto Takahashi, Sudarshan Sharma, Fabian Jerzembeck, Stephen J. Blundell, Graeme M. Luke, J. C. Séamus Davis, Jonathan Ward

**Affiliations:** ^a^ Department of Physics, University College Cork, Cork T12 R5C, Ireland; ^b^ Clarendon Laboratory, Oxford University, Oxford OX1 3PU, United Kingdom; ^c^ Department of Physics, McMaster University, Hamilton, ON L8S 4L8, Canada; ^d^ Max-Planck Institute for Chemical Physics of Solids, Dresden D-01187, Germany

**Keywords:** vitrification, dynamical heterogeneity, supercooled liquids, spin-ice, emergent magnetic monopoles

## Abstract

Dynamical heterogeneity, in which transitory local fluctuations occur in the conformation and dynamics of constituent particles, is widely hypothesized to be essential to the evolution of supercooled liquids into the structural glass state. Yet its microscopic spatiotemporal phenomenology is challenging to detect directly in molecular glass forming liquids. Because recent theoretical advances predict that corresponding dynamical heterogeneity could occur in supercooled magnetic monopole fluids (Proc. Nat. Acad. Sci. 112, 8549 (2015)), we searched for such phenomena in Dy_2_Ti_2_O_7_. By measuring its microsecond-resolved spontaneous magnetization fluctuations M(t,T) we detected a sharp bifurcation in monopole noise characteristics below T≈1,500 mK, with the appearance of powerful spontaneous monopole current bursts. This intense dynamics emerges upon entering the supercooled monopole fluid regime, reaches maximum strength near T≈750 mK and then collapses along with coincident loss of ergodicity approaching $T_{g}\approx 250\;\mathrm{mK}$. Moreover, when the four-point dynamical susceptibility $\chi_{4}\left(\tau ,T\right)$ is determined directly from temperature dependence of correlations in M(t,T), it evolves as predicted when dynamical heterogeneity is present, revealing its simultaneously and rapidly escalating length and time scales, ξ(T) and $\tau_{4}\left(T\right)$. This overall phenomenology greatly expands our empirical knowledge of supercooled monopole fluids and, more generally, demonstrates techniques for detection of the time sequence, magnitude, statistics, and correlations of dynamical heterogeneity, access to which may greatly accelerate fundamental vitrification studies.

## Significance

Glasses are ubiquitous, yet their microscopic mechanism remains unidentified. A key hypothesis is that supercooled liquids evolve into glasses through spatiotemporal dynamical heterogeneity i.e. transitory local fluctuations in the conformation and dynamics of constituent particles. While four-point dynamic correlation functions could validate such models, these are challenging to detect directly in glass-forming liquids. Because analogous physics may exist for monopoles in spin ice, we searched for dynamical heterogeneity in the supercooled monopole fluid of Dy_2_Ti_2_O_7_. By measuring monopole noise spectra to estimate the monopole four-point correlation function, we demonstrate that supercooled monopoles exhibit numerous signatures anticipated for dynamical heterogeneity. This highlights both a striking universality in vitrification dynamics and how spin ice offers unique opportunities for dynamical heterogeneity studies.

“*The deepest and most interesting unsolved problem in solid state theory is probably the theory of the nature of glass and the glass transition*” P. W. Anderson (1). Although most pure liquids crystallize at their melting temperature, glass-forming liquids instead first enter the supercooled state (2, 3, 4) and eventually transition into a glass state. During this evolution, it is widely hypothesized that the dynamics of constituent particles slow down radically and in an increasingly heterogeneous fashion (2–7) so that local regions relax on different trajectories at different rates in a continuously evolving yet globally ergodic fashion. These phenomena are thermally activated (8–13) events about an unchanging thermodynamic equilibrium. How their atomic-scale phenomenology controls the vitrification process remains an intense focus of modern research (2–16). Current theoretical progress includes predictions of frequency-resolved loss of ergodicity (14); of trapped nanoscale droplets with internal fluidic particle dynamics (15); and of evolution from supercooled dynamical heterogeneity through the glass transition (16). Only recently, however, have such phenomena been hypothesized to occur (17–21) upon cooling the magnetic monopole fluids of spin-ice.

The most pertinent material is Dy_2_Ti_2_O_7_ which contains a sublattice of corner-sharing tetrahedra, each having a magnetic Dy^3+^ ion at its four vertices. The Dy magnetic moments ($\mu \approx 10\mu_{B}$) are Ising-like, being constrained to point along their local [111] directions towards or away from the tetrahedron center. The consequent dipolar spin-ice Hamiltonian is (22)

[1] $$\begin{array}{c} H=-J\sum_{\left\langle \mathrm{ij}\right\rangle }S_{i}\cdot S_{j}+Da^{3}\sum_{i<j}\left(\frac{S_{i}\cdot S_{j}}{{\left|r_{\mathrm{ij}}\right|}^{3}}-\frac{3\left(S_{i}\cdot r_{\mathrm{ij}}\right)\left(S_{j}\cdot r_{\mathrm{ij}}\right)}{{\left|r_{\mathrm{ij}}\right|}^{5}}\right). \end{array}$$

Here $S_{i}$ represent the Ising spin at each Dy site, $r_{\mathrm{ij}}$ are the intersite distances, J≈1.1 K is the exchange energy, $D=\mu_{0}\mu^{2}/\left(4\pi a^{3}\right)$ the nearest-neighbor dipole interaction energy, and a is the nearest-neighbor distance between moments. From Eq. **1**, only six possible ground-state spin configurations exist on each tetrahedron, all being 2-in/2-out spin arrangements (23). Although the dipole interactions in Eq. **1** could (24) generate a first-order phase transition to a long-range magnetic ordered state, no signature of such a state has ever been observed to temperatures below T≈50 mK (25). Hence, the monopole kinetics in spin-ice as T→0 also remain a focus of concentrated research (17–21).

## Supercooling the Monopole Fluid

By contrast, the excited states governed by Eq. **1** at higher temperatures T≳1.5 K, are well understood (26–28) to be mobile magnetic charges (monopoles) of both signs: +m for 1-in:3-out and -m for 3-in:1-out (*SI Appendix*, section I). They exist in a magnetic-charge neutral fluid in which equal numbers of *+m* and *-m* are thermally excited across the Dy spin-flip energy barrier Δ≈4.3 K. However, below T≈1.5 K this monopole fluid enters a supercooled state (29). Here, the magnetic susceptibility χ(ω,T) exhibits a Havriliak-Negami (HN) form (29, 30) characteristic of supercooled glass forming liquids. Further, the susceptibility-derived relaxation time $\tau_{\chi }\left(T\right)=A\exp((DT_{g})/((T-T_{g})))$ where D is the fragility index, evolves with $T_{g}\approx 240\;\mathrm{mK}\pm 30\;\mathrm{mK}$ on a Vogel–Tammann–Fulcher (VTF) trajectory (29) characteristic of supercooling (*SI Appendix*, section II). Additionally, Monte Carlo simulations (31) predicting magnetization noise with spectral density $S_{M}\left(\omega ,T\right)\propto \tau_{N}\left(T\right)/\left(1+\left({\omega \tau_{N}\left(T\right)}^{b}\right)\right)$ led to the discovery (32) of magnetic monopole noise exhibiting a power-law exponent b(T)≈1.5 approaching T≈1 K and noise-derived relaxation time $\tau_{N}\left(T\right)$ evolving on an equivalent VTF trajectory (32–34). Because this is consistent with advanced monopole transport theories based on fractal clusters of monopole trajectories (19), heterogeneous monopole dynamics is construed. Altogether, the observed broad distribution of χ(ω,T) relaxation times $\tau_{\chi }\left(T\right)$ (29), the VTF form evidenced by $\tau_{\chi }\left(T\right)$ (29), and the monopole noise power-law b(T) (32), imply by analogy with general supercooled glass-forming liquids that monopole dynamical heterogeneity should exist in Dy_2_Ti_2_O_7_.

Microscopic theories (17–21) have long focused on frustrated monopole kinetics approaching the T→0 state of spin-ice. Typically, the high-temperature state is viewed as a thermally activated plasma of quasi-free (anti)monopoles (26–28) (state I). Refrigeration from state I is anticipated to yield a supercooled monopole fluid (29) (state II) potentially sustaining some form of dynamical heterogeneity (19–21). The ultralow temperature state of spin ice (state III) remains empirical terra incognita. For example, extended spin-ice models predict growing dynamical heterogeneity resulting in loss of ergodicity near T/J≈0.1 when spin–spin correlation time evolves (17). Similarly, dumbbell spin-ice models predict that enhancing dynamical heterogeneity near T≈400 mK in Dy_2_Ti_2_O_7_, should cause the fluctuation–dissipation ratio $\omega S_{M}\left(\omega ,T\right)/T\chi^{\prime \prime }\left(\omega ,T\right)$ to evolve from its ergodic high-temperature limit (20). However, the empirical phenomenologies of monopole dynamics in states II and III of spin-ice are largely unexplored. Recent theoretic advances actualize these concepts by predicting a new form of heterogeneous monopole dynamics based on the existence of two spin-dynamical time-scales, constraining the trajectories of monopoles to nanoscale clusters (19), and this hypothesis is supported indirectly by recent experiments (32–34).

If its microscopic kinetics were not frustrated, dipolar spin ice is predicted to undergo a first order transition to a magnetically long-range ordered phase (24). Yet this ordered state has never been observed in Dy_2_Ti_2_O_7_ or Ho_2_Ti_2_O_7_ apparently due to frustration of the necessary global spin reconfigurations by a network of Dirac-strings threading the material, each string being due to passage of a magnetic (anti)monopole. The consequent spin ice “clusters” are conceived as local regions of 2-in:2-out spin configurations that have net coarse-grained magnetization. Within each, the motion of (anti)monopoles flips one spin per tetrahedron all along its Dirac-string trajectory, resulting in a different configuration of 2-in:2-out spins due to the passage of each monopole. However, once all internal monopole motion has halted, the net magnetic moment of such a cluster is unchanged. By definition, such a reconfiguration of 2-in:2-out spin arrangements within a spin ice cluster corresponds to a burst of monopole motion. Spin ice theories describe that process colloquially as e.g. “large-scale annealing events where many monopoles exchange position in bursts and the effective potential landscape changes akin to an avalanche” (21). Fundamentally, monopole current bursts and spin ice cluster reconfigurations are synonyms for the same microscopic concept: One cannot have the reorganization of the underlying 2-in:2out spin patterns (the spin ice cluster configuration) without the simultaneous movement of the monopoles (the monopole current).

## Coterminous Monopole Noise and Magnetic Susceptibility

**Fig. 1.**
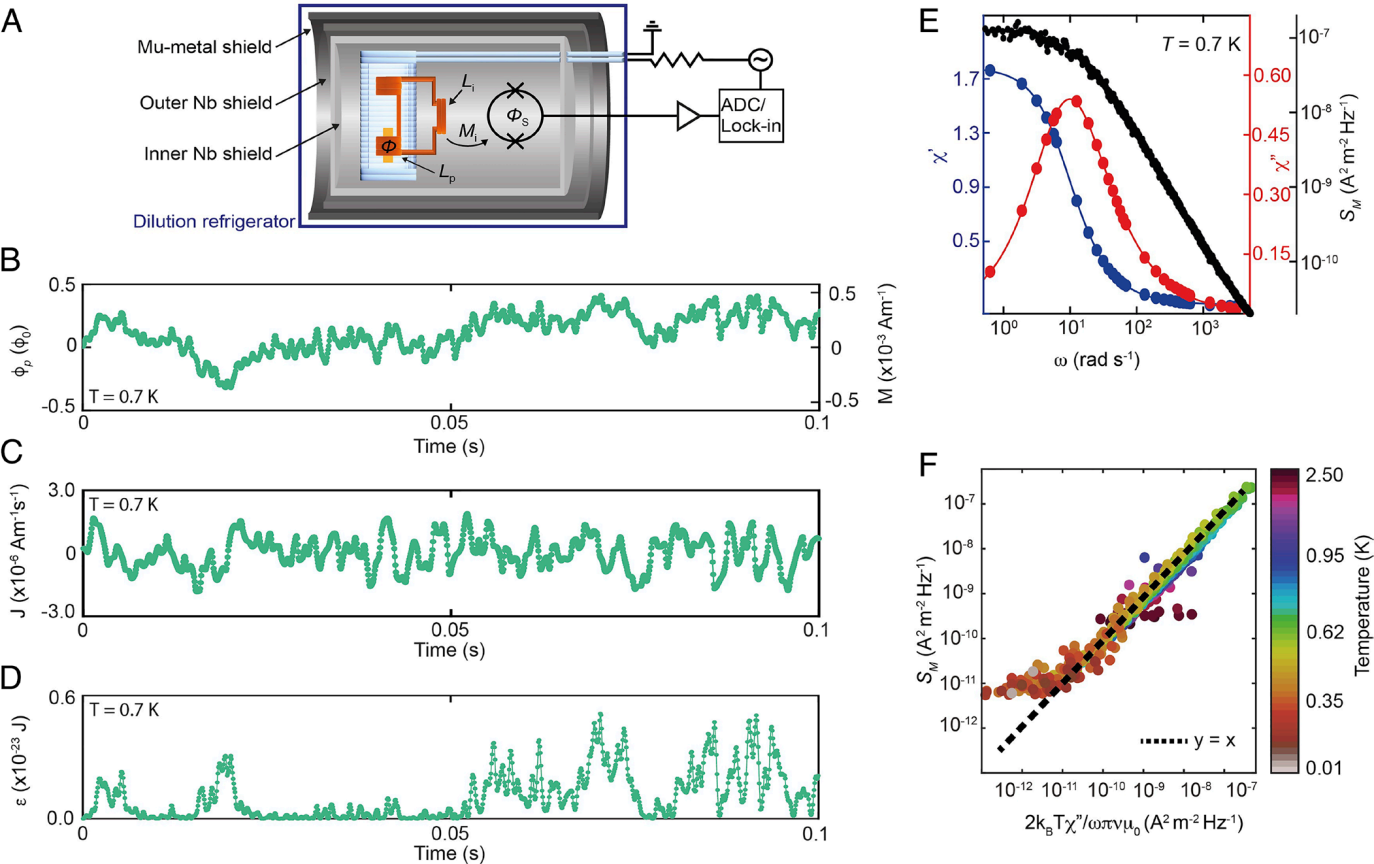
Magnetic monopole noise spectrometry. (*A*) Schematic of the experimental apparatus we use for detection of dynamical heterogeneity due to magnetic monopole current bursts in the supercooled monopole fluid of Dy_2_Ti_2_O_7_. (*B*) Typical example of the magnetic flux $\Phi_{p}\left(t,T\right)$ generated spontaneously by Dy_2_Ti_2_O_7_ samples. The time dependence of sample magnetization along the symmetry axis of the superconductive pickup coil is then $M\left(t,T\right)=\Phi_{p}\left(t,T\right)/{A\mu }_{0}$ from Eq. **3**, as shown on RHS. (*C*) Typical time dependence of magnetic monopole current ${J\left(t,T\right)\equiv \dot{\Phi }}_{p}\left(t,T\right)/\mu_{0}$ along the axis of the pickup coil from Eq. **4**. (*D*) Typical total magnetic energy $\varepsilon \left(t,T\right)\equiv \Phi_{p}^{2}\left(t,T\right)/{2L}_{p}$ due to the monopole currents within the sample as detected by the pickup coil at any instant t, from Eq. **5**. (*E*) The power spectral density of the Dy_2_Ti_2_O_7_ sample magnetization fluctuations $S_{M}\left(\omega ,T\right)\equiv \lim_{P\to \infty }\frac{1}{P}{\left|\int_{-\frac{P}{2}}^{\frac{P}{2}}M\left(t,T\right)e^{i\omega t}dt\right|}^{2}$, where P is the time period over which the time sequence of $M\left(t,T\right)$ is recorded as in B. Typical Dy_2_Ti_2_O_7_ magnetic susceptibility $\chi^{\prime }\left(\omega ,T\right),\chi^{\prime \prime }\left(\omega ,T\right)$ and magnetization noise spectrum $S_{M}\left(\omega ,T\right)$ measured coterminously at T=700 mK. (*F*) Temperature dependence of coterminous $S_{M}\left(\omega ,T\right)$ and $\chi^{\prime \prime }\left(\omega ,T\right)2kT/\omega \pi \upsilon \mu_{0}$. Evidently, monopole ergodicity parameterized by $X\left(\omega ,T\right)\equiv S_{M}\left(\omega ,T\right)/{\{\chi }^{\prime \prime }\left(\omega ,T\right)2kT/\omega \pi \upsilon \mu_{0}\}$ diminishes slowly beginning near T≈500 mK, to be lost manifestly by T≲250 mK.

To explore such concepts in Dy_2_Ti_2_O_7_, we use SQUID-based flux-noise spectrometry with magnetic field sensitivity $\delta B{=\mu }_{0}\delta M{\leq 10}^{-14}T/\sqrt{Hz}$, where $\mu_{0}$ is the permeability of vacuum, shown schematically in Fig. 1*A*. Here, $L_{p}$ is the inductance of both the sample pickup coil and of a counter wound compensation coil, $L_{i}$ is a SQUID-input coil inductance, and $M_{i}$ is a mutual inductance to SQUID. Our spectrometer is operated on a cryogen-free dilution refrigerator in the range 15 mK≲T≲2,500 mK. The time sequence of the magnetic flux generated by the sample, $\Phi_{p}\left(t,T\right)$, is measured at each temperature T with microsecond precision, using a persistent superconducting circuit that transforms it into the flux Φ(t,T) at the SQUID input coil

[2] $$\Phi \left(t,T\right)=(M_{i}/(2L_{p}+L_{i}))\Phi_{p}\left(t,T\right)=\Phi_{p}\left(t,T\right)/\beta ,$$

where β is a precisely and independently known calibration constant of the instrument (*SI Appendix*, section III). Although the procedures for analyzing magnetic monopole noise from time sequences of magnetic flux $\Phi_{p}\left(t,T\right)$ are well established (32–34), we review them here for didactic purposes. As illustrated in Fig. 1*B*, the time dependence of sample magnetization along the symmetry axis of the superconductive pickup coil is

[3] $$M\left(t,T\right)\equiv \Phi_{p}\left(t,T\right)/A\mu_{0},$$

where A is the sample cross-sectional area. The time dependence of the component of magnetic monopole current J(t,T) (Fig. 1*C*) along the same axis of the pickup coil is

[4] $$J(t,T)\equiv {\dot{\Phi }}_{p}\left(t,T\right)/\mu_{0}.$$

The total magnetic energy ε(t,T) (Fig. 1*D*) due to the monopole currents which is stored in the pickup coil at any instant is

[5] $$\varepsilon (t,T)\equiv \Phi_{p}^{2}\left(t,T\right)/2L_{p}.$$

The power spectral density of the magnetization fluctuations from Eq. **3** is

[6] $$S_{M}(\omega ,T)\equiv \lim_{P\to \infty }\frac{1}{P}{\left|\int_{-\frac{P}{2}}^{\frac{P}{2}}M\left(t,T\right)e^{i\omega t}dt\right|}^{2},$$

where P is the time period over which the time sequence of M(t,T) is recorded. The noise contribution of the superconductive circuitry and SQUID as measured separately absent the sample, are first subtracted to yield the sample $S_{M}\left(\omega ,T\right)$. Finally, the magnetic susceptibility χ(ω,T) is the magnetic response of the sample to an applied field H at angular frequency ω. The in-phase and out-of-phase response of magnetic flux from the sample only, $\Phi_{p}^{X}\left(\omega ,T\right)$; $\Phi_{p}^{Y}\left(\omega ,T\right)$, as measured using a calibrated lock-in amplifier to analyze $\Phi_{p}\left(t,T\right)$, yields the in-phase and out-of-phase susceptibility as

[7] $$\begin{array}{c} \chi \left(\omega ,T\right)\equiv \chi^{\prime }\left(\omega ,T\right)+i\chi^{\prime \prime }\left(\omega ,T\right)\\ =\frac{1}{\mathrm{NA}}\left[\frac{\Phi_{p}^{X}\left(\omega ,T\right)}{\mu_{0}H}+i\frac{\Phi_{p}^{Y}\left(\omega ,T\right)}{\mu_{0}H}\right] \end{array}$$

where N is the number of turns in the superconductive pickup coil. This magnetic susceptibility χ(ω,T) is measured coterminously with the magnetization noise $S_{M}\left(\omega ,T\right)$, using identical samples/detectors, over the temperature range 250 mK<T<2,500 mK 2,500 mK. As temperature falls, the monopole/antimonopole pairs become dilute (*SI Appendix*, section I) with approximate density $n\approx n_{0}exp\left(-\Delta /kT\right)$ where $n_{0}=1/a_{d}{\approx 10}^{28}m^{-3}$ and Δ≈4.3 K. Thus at T≈250 mK, $n\approx 10^{20}m^{-3}$ so that for sample volume $\upsilon \approx 10^{-8}m^{3},n{\approx 10}^{12}$ pairs remain. This number is well above our detection threshold. All the above analysis procedures are exemplified in Fig. 1*C*, *D*, based on a typical time sequence of flux noise $\Phi_{p}\left(t,T\right)$ in Fig. 1*B*, and all subsequent studies reported below are based on such data.

For an ergodic monopole fluid, the fluctuation–dissipation theorem (FDT) linking $S_{M}\left(\omega ,T\right)$ to the imaginary magnetic susceptibility $\chi^{\prime \prime }\left(\omega ,T\right)$ would predict (20)

[8] $$S_{M}(\omega ,T)=2k_{B}T\chi^{\prime \prime }\left(\omega ,T\right)/\omega \pi \upsilon \mu_{0},$$

where υ is the sample volume, $k_{B}$ is Boltzmann’s constant and we use SI units throughout. Conversely, a violation of FDT would imply a loss of ergodicity in the monopole fluid. For our Dy_2_Ti_2_O_7_ samples, a typical set of coterminously measured $\chi^{\prime }\left(\omega ,T\right)$, $\chi^{\prime \prime }\left(\omega ,T\right)$ and $S_{M}\left(\omega ,T\right)$ are plotted in Fig. 1*E* (*SI Appendix*, section IV). Here, because of the wide distribution of microscopic relaxation times (29), even when $\tau_{\chi }\left(T\right)$ increases to the glass transition at $T_{g}\approx 250\;\mathrm{mK}$ (29), high frequency monopole dynamics must still be present at a subset of sites. Hence, to explore the evolution of Eq. **8** to lowest temperatures, we plot in Fig. 1*F* the measured $S_{M}\left(\omega ,T\right)$ versus independently measured $2k_{B}T\chi^{\prime \prime }\left(T\right)/\omega \pi \upsilon \mu_{0}$ at frequencies where dynamics is manifestly occurring in the monopole noise. Evidently, the fluctuation–dissipation theorem for the magnetic monopole fluid holds for T≳500 mK. However, because of the observed collapse of $\begin{array}{c} X\left(\omega ,T\right)\equiv S_{M}\left(\omega ,T\right)\omega \pi \upsilon \mu_{0}/2k_{B}T\chi^{\prime \prime }\left(\omega ,T\right) \end{array}$$\begin{array}{c} 2k_{B}T\chi^{\prime \prime }\left(\omega ,T\right) \end{array}$ from X=1 starting below T≲500 mK, the monopole fluid here slowly exits the ergodic regime. Eventually, FDT is strongly violated with complete loss of monopole ergodicity at T≲250 mK as shown Fig. 1*F* (*SI Appendix*, section V).

## Discovery of monopole dynamical heterogeneity

**Fig. 2.**
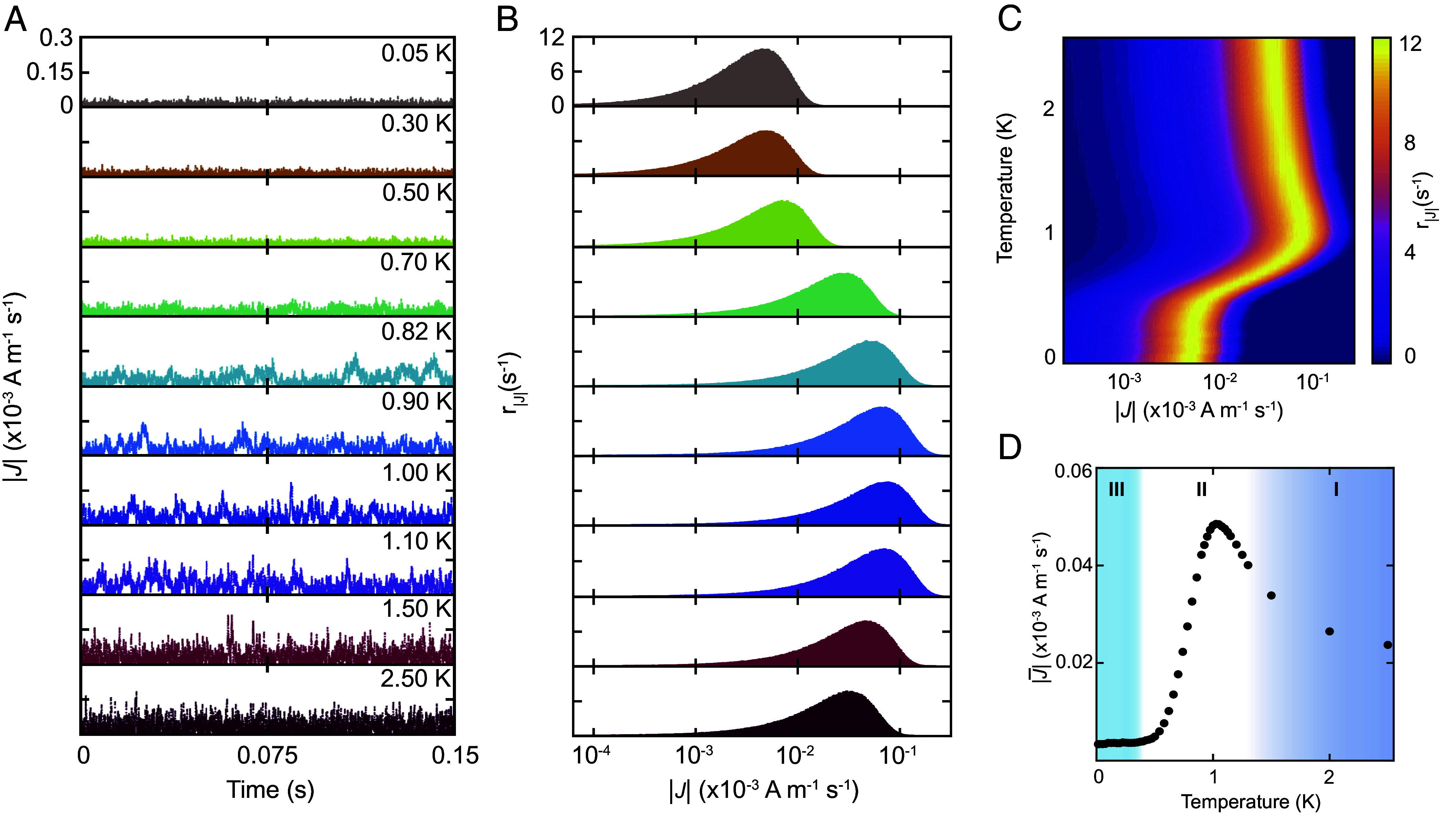
Monopole current bursts in the supercooled state. (*A*) Typical measured time sequences of monopole current magnitudes |J(t,T)| from Eq. **4**, over a wide range of temperatures spanning the homogeneous monopole fluid regime I, into the supercooled regime II, and finally the T→0 regime III. (*B*) Typical measured probability distribution of the monopole current burst magnitudes |$J\left(t,T\right)|=\left|\dot{\Phi }\left(t,T\right)/\mu_{0}\right|$ derived directly from the unprocessed and non-normalized $\Phi \left(t,T\right)$ data, e.g., in A. The measured monopole currents span an intensity range of approximately five orders of magnitude, with maximum intensity individual events occurring at T≈900 mK. These data are highly typical of multiple Dy_2_Ti_2_O_7_ samples studied. (*C*) Typical per unit time rate $r_{\left|J\right|}$ of monopole currents having magnitude |J|, measured versus temperature T. The rate of occurrence $r_{\left|J\right|}$ of a monopole current with magnitude |J| is defined as the number η(|J|) observed in given time interval P: $r_{\left|J\right|}\equiv \eta \left(|J|\right)/P.$ (*D*) Average measured intensity of monopole current bursts $\bar{\left|J\right|}$ versus temperature. Clearly, approaching the supercooled regime below T≈1500 mK they intensify dramatically, only to fall precipitously reaching a plateau T≲250 mK.

A key signature of the dynamical component of monopole dynamical heterogeneity would be random and intense monopole current bursts (19, 21). Hence, we next measure the time-sequences of flux threading the sample at its pickup coil, $\Phi_{p}\left(t,T\right)$, typically for a continuous period of P=1000 seconds. If each monopole exhibits a magnetic charge m and total magnetic flux $\Phi_{m}=m\mu_{o}$ (26) and because the magnetic flux through any superconductive closed-loop circuit is quantized, when a magnetic monopole passes through such a loop it generates a supercurrent exactly counterbalancing $\Phi_{m}$. This is detectable by a SQUID as a flux generated elsewhere in the circuit. Under these circumstances, the time dependence of net monopole current through the pickup coil is ${J\left(t,T\right)\equiv \dot{\Phi }}_{p}\left(t,T\right)/\mu_{0}$ (*SI Appendix*, section VI). For such measurements of J(t,T) derived from the unprocessed $\Phi_{p}\left(t,T\right)$ data, we use an 80μs box-car average, with an exemplary time sequence of J(t,T) as derived from $\Phi_{p}\left(t,T\right)$ in Fig. 1*B*, being shown in Fig. 1*C*. Typical measured time sequences of monopole current magnitudes |J(t,T)| derived using Eq. **4**, over a wide range of temperatures traversing from the homogeneous monopole fluid regime into the supercooled regime, are shown in Fig. 2*A*. The measured probability distribution, $r_{\left|J\right|}$, of the magnitudes of monopole currents |J(t,T)| during P=1000 second periods, is given by the number η(|J|) of monopole currents per unit time having magnitude |J|: $r_{\left|J\right|}=\eta \left(|J|\right)/P$. A typical set of distributions of $r_{\left|J\right|}$ is then shown in Fig. 2*B*. Here, the monopole current magnitudes range in intensity over almost five orders of magnitude with maximum intensity occurring near T=1 K. The evolution of $r_{\left|J\right|}$, is presented in Fig. 2*C* versus T. The time-averaged intensity of these same monopole currents $\bar{\left|J\right|}\left(T\right)$ is shown in Fig. 2*D*.

**Fig. 3.**
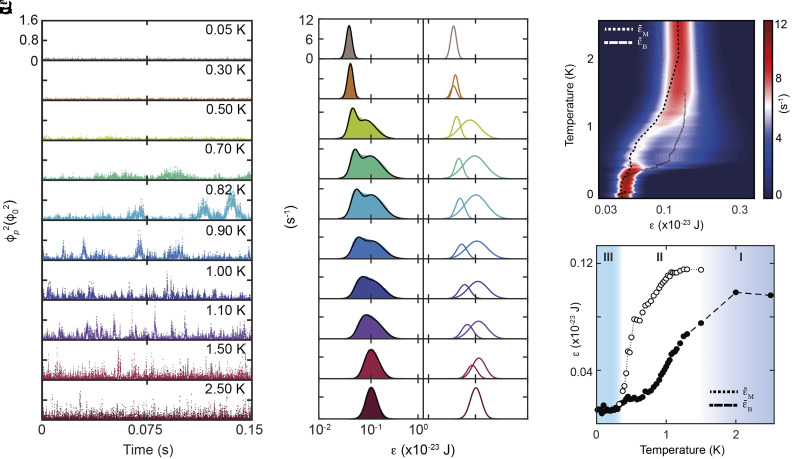
Monopole noise bifurcation due to dynamical heterogeneity. (*A*) Typical examples of the $\Phi_{p}^{2}\left(t,T\right)$ from directly measured time dependence of spontaneous magnetic flux $\Phi_{p}\left(t,T\right)$. This is shown, for example, at temperatures 50 mK, 300 mK, 500 mK, 700 mK, 900 mK, 1,500 mK, and 2,500 mK. (*B*) Typical histograms of the measured rate of flux states $r_{\varepsilon }\left(T\right)$ versus ε. We define the rate of occurrence $r_{\varepsilon }\left(T\right)$ of any state with energy ε as the number m(ε) observed in given time interval *P*: $r_{\varepsilon }\equiv m\left(\varepsilon \right)/P.$ Conventional monopole generation-recombination noise with a simple Gaussian distribution persists until T≈1500 mK. More intense monopole current bursts with far higher energy appear below this temperature resulting in a bimodal distribution of probabilities as shown via histograms (*Left*), and by the fit curves to each histogram shown (*Right*). Eventually below T≲250 mK, the bimodal distribution of monopole current burst energies disappears. (*C*) Monopole noise bifurcation effect in Fig. 3*B* is presented as a color-coded 2D histogram containing $r_{\varepsilon }$ versus T. Dashed curve ${\bar{\varepsilon }}_{M}\left(T\right)$ indicates the average energy of conventional monopole noise, while the dotted curve ${\bar{\varepsilon }}_{B}\left(T\right)$ plots the average energy of monopole current bursts ascribed to dynamical heterogeneity. (*D*) Relative intensities of average energy of monopole current bursts ${\bar{\varepsilon }}_{B}\left(T\right)$ and of conventional monopole noise ${\bar{\varepsilon }}_{M}\left(T\right)$.

As to the energetics of these phenomena: Fig. 1*B* provides a typical example of magnetization fluctuations in terms of $\Phi_{p}\left(t,T\right)$ and the magnetic energy ε associated with each global monopole configuration can then be determined accurately from the unprocessed $\Phi_{p}\left(t,T\right)$ signal. From elementary superconductive circuit analysis, $\varepsilon \left(t,T\right)\equiv \Phi_{p}^{2}\left(t,T\right)/{2L}_{\text{p}}$ (*SI Appendix*, section VI). Typical examples of measured values of $\Phi_{p}^{2}\left(t,T\right)$ during 1,000 s periods are shown in Fig. 3*A* for a representative set of temperatures. The measured probability distribution $r_{\varepsilon }\left(T\right)$, of global monopole dynamic events with energy ε per unit time, is given by the number $m\left(\varepsilon \right)$ of times a given energy ε occurs in the continuous energy signal: $r_{\varepsilon }=m\left(\varepsilon \right)/P$. Typical histograms of $r_{\varepsilon }\left(T\right)$ are presented in Fig. 3*B* versus *T.* Strikingly, while the energetics $\varepsilon \left(t\right)$ are Gaussian and narrow in distribution for T≳1,500 mK, at lower temperatures a sharp bifurcation occurs into a bimodal distribution containing less frequent highly energetic events, each exemplifying a monopole-current burst. Eventually below T≲250 mK, these phenomena disappear, and a single low-energy Gaussian distribution reappears. This complete phenomenology is represented by all measured $r_{\varepsilon }\left(T\right)$ data shown as a color-coded 2D histogram in Fig. 3*C*. Here, the dashed curve ${\bar{\varepsilon }}_{M}\left(T\right)$ indicates the average energy of conventional monopole generation-recombination noise (32–34) while the dotted curve ${\bar{\varepsilon }}_{B}\left(T\right)$ plots the average energy of monopole current bursts ascribed to dynamical heterogeneity. The measured relative energy intensities of monopole current bursts ${\bar{\varepsilon }}_{B}\left(T\right)$ and of ${\bar{\varepsilon }}_{M}\left(T\right)$ are shown in Fig. 3*D*. Clearly, there are two populations of monopole currents: those related to conventional monopole noise (32–34), plus more intense current bursts existing over extended time periods and producing large excursions in $\Phi_{p}\left(t,T\right)$. A strong maximum in monopole current burst intensity occurs entering the supercooled regime, followed by a rapid collapse below T≲500 mK (Fig. 2*D*). The ostensible cause of this bimodality in monopole currents is that elementary monopole generation recombination processes (32–34) fall into one class of monopole dynamics, whereas the monopole current bursts causing dynamical reconfiguration of larger spin ice clusters represents a far more intense second class (19, 21).

The relaxation time of magnetization fluctuations $\tau_{N}\left(T\right)$ in the supercooled regime of Dy_2_Ti_2_O_7_ is determined via its power spectral density $S_{M}\left(\omega ,T\right)$, by fitting to $S_{M}\left(\omega ,T\right)\propto \tau_{N}\left(T\right)/$$\left(1+{(\omega \tau_{N}\left(T\right)}^{b}\right)$ over almost four orders of magnitude in $\tau_{N}\left(T\right)$ (*SI Appendix*, section VII). As had previously been established from susceptibility studies (29), here we find that $\tau_{N}\left(T\right)$ for Dy_2_Ti_2_O_7_ is governed demonstrably by the Vogel-Tammann-Fulcher (VTF) equation $\tau_{N}\left(T\right)=Aexp\left(DT_{g}/T-T_{g}\right)$, with fragility D≈14 and a well-defined glass temperature $T_{g}≅240$ mK (*SI Appendix*, section VII), while sustaining ergodicity at all higher temperatures. Equivalent relaxation time characteristics evolving according to the VTF equation are widely observed in molecular glass-forming liquids (2–8).

Finally, while the dynamical nature of the monopole current bursts is self-evident (Figs. 1–3) their heterogeneity requires quantification. In the general theory of dynamical heterogeneity in supercooled liquids (2–8), slower dynamics continuously transform to faster dynamics and vice versa at ever-changing nanoscale regions with correlation length ξ(T), a spatial scale that increases rapidly towards the glass transition. The empirical challenge for monopole fluid studies is then to characterize such coterminous phenomena in terms of their lengthening lifetimes (which are well established for Dy_2_Ti_2_O_7_ (28, 29, 32)), and of their increasing length scales ξ(T) which are unknown. In principle, the latter may be determined by using the four-point susceptibility $\chi_{4}\left(\tau ,T\right)$ (35–37), a measure of the fluctuations in the two-point correlation function. Theories of glass formation report that $\chi_{4}\left(\tau ,T\right)$ typically exhibits a strong maximum in relaxation time τ dependence, whose height is proportional to the volume containing the correlated motion of molecules (38–41). Direct experimental measurements of $\chi_{4}\left(\tau ,T\right)$ have focused on non-thermodynamic colloidal and granular materials (42–44) where heterogeneity is accessible experimentally by imaging. But direct measurements of $\chi_{4}\left(\tau ,T\right)$ are very challenging for physical systems containing an ensemble of nanoscale particles in thermodynamic equilibrium, e.g., supercooled glass-forming molecular liquids.

To circumvent this limitation, another approach has been developed (13, 37, 45, 46). Given a time-series of measurements $A\left(t,T\right)$, where A is a property of a system in thermal equilibrium at temperature *T*, the standard two-point correlation function is $C_{A}\left(t,\tau ,T\right)=A\left(t,T\right)A\left(t+\tau ,T\right)$ while the consequent autocorrelation function is $F_{A}\left(\tau ,T\right)\equiv {\left\langle A\left(t,T\right)A\left(t+\tau ,T\right)\right\rangle }_{t}$ (*SI Appendix*, section VIII). The dynamic susceptibility $\chi_{4}\left(\tau ,T\right)$ can then be estimated (13, 37, 45, 46) from the response function $\chi_{T}\left(\tau ,T\right)$ to temperature variations which is defined as

[11] $$\chi_{T}\left(\tau ,T\right)=\partial F_{A}(\tau ,T)/\partial T.$$

When the fluctuation–dissipation relation is valid, it has then been established (45) that $\begin{array}{c} k_{B}T^{2}\chi_{T}\left(\tau ,T\right)=N \end{array}$ $\begin{array}{c} {\left\langle \delta C_{A}\left(t,\tau ,T\right)\delta H\left(t,0,T\right)\right\rangle }_{t} \end{array}$ where $\begin{array}{c} \delta C_{A}\left(t,\tau ,T\right)=C_{A}\left(t,\tau ,T\right)- \end{array}$ $\begin{array}{c} {\left\langle C_{A}\left(\tau ,T\right)\right\rangle }_{t} \end{array}$ is the fluctuation of $C_{A}\left(t,\tau ,T\right)$ about its mean value ${\left\langle C_{A}\left(\tau ,T\right)\right\rangle }_{t}$, while δH(t,0,T) is the fluctuating enthalpy per particle, and N is the total number of particles. Experimental and numerical studies (37, 45) have shown that from these concepts it follows

[12] $$\chi_{4}\left(\tau ,T\right)\approx k_{B}T^{2}c_{p}{\left(T\right)}^{-1}{\left[\chi_{T}\left(\tau ,T\right)\right]}^{2},$$

where $c_{p}\left(T\right)$ is the specific heat capacity of the particles undergoing vitrification. In the context of Dy_2_Ti_2_O_7_ magnetic monopole fluids where $\Phi_{p}\left(t,T\right)$ is the thermodynamic property fluctuating in time, the correlation function and autocorrelation functions have been established previously (32): The relevant two-point correlation function is $C_{\Phi }\left(t,\tau ,T\right)=\Phi_{p}\left(t,T\right)\Phi_{p}\left(t+\tau ,T\right)$ while its autocorrelation function is $F_{\Phi }\left(\tau ,T\right)\equiv {\left\langle \Phi_{p}\left(t,T\right)\Phi_{p}(t+\tau ,T)\right\rangle }_{t}$. The four-point dynamic susceptibility $\chi_{4}\left(\tau ,T\right)$ of the monopole fluid can then be estimated using Eq. **12**. To explore this concept, the normalized autocorrelation function $F_{\Phi }\left(\tau ,T\right)$ is calculated from the unprocessed $\Phi_{p}\left(t,T\right)$ data (in a P=1000 second interval) as

**Fig. 4.**
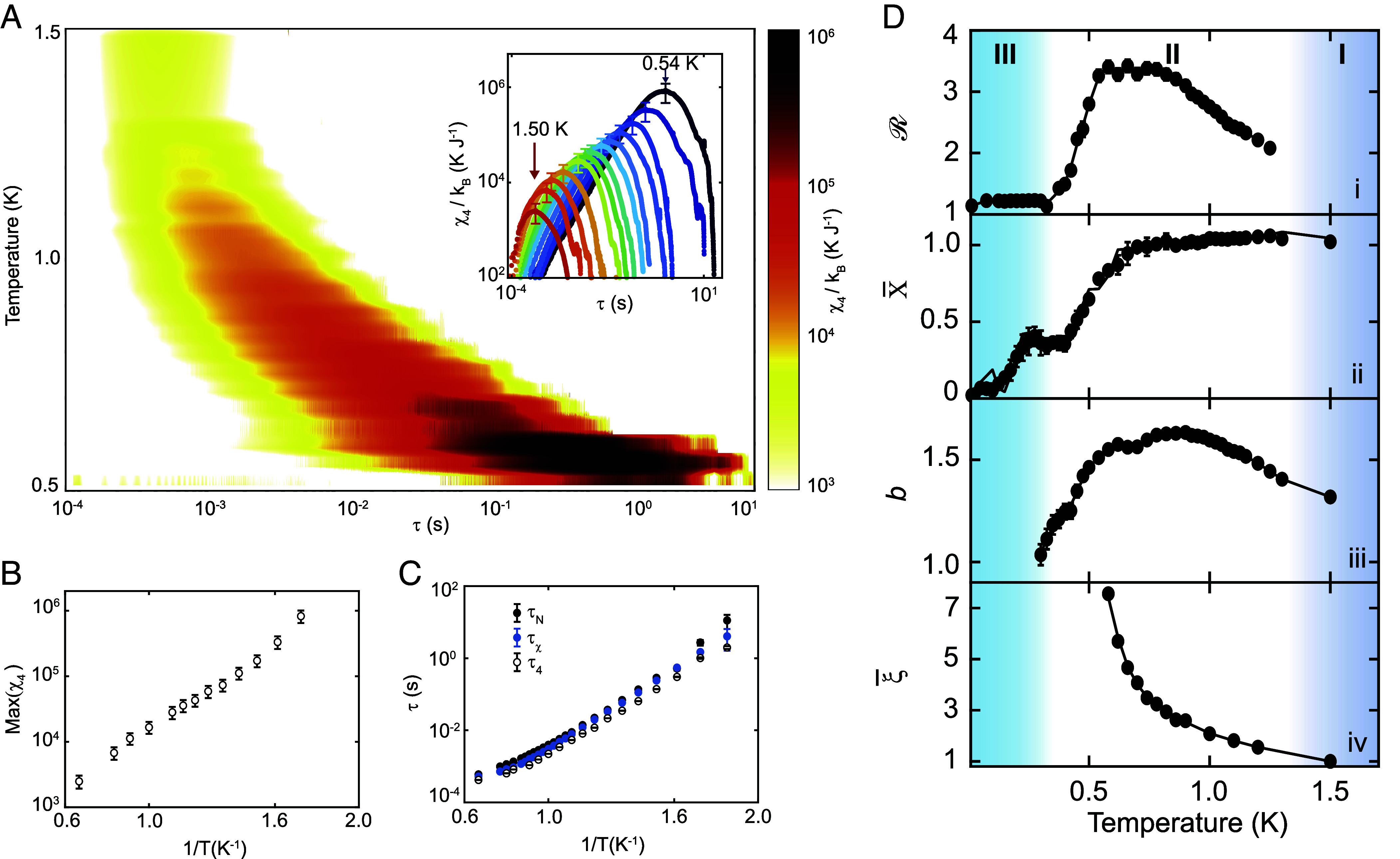
Measured $\chi_{4}(\tau ,T)$ and $\bar{\xi }(T)$ of monopole dynamical heterogeneity. (*A*) Measured dynamical susceptibility $\chi_{4}\left(\tau ,T\right)$ of the supercooled monopole fluid of Dy_2_Ti_2_O_7_. Inset: $\chi_{4}\left(\tau ,T\right)$ shown on a log–log scale at a representative set of temperatures. (*B*) Evolution of ${Max(\chi }_{4}\left(\tau ,T)\right)$ with temperature shows the striking growth in relative correlation length of dynamical heterogeneity in Dy_2_Ti_2_O_7._ (*C*) Evolution of the relaxation times with inverse temperature 1/T. The filled black circles represent $\tau_{N}\left(T\right)$ extracted by fitting the noise spectra $S_{M}.$ The blue filled circles represent $\tau_{\chi }\left(T\right)$ which is extracted as the peak of the fit to the imaginary magnetic susceptibility. The empty circles represent $\tau_{4}\left(T\right)$ extracted from the time at which dynamical susceptibility $\chi_{4}\left(T\right)$ achieves its maximum. (*D*) (*i*) Measured ratio of maximal monopole current bursts relative to the conventional magnetic monopole noise $R\equiv Max(\varepsilon_{B})/\overset{¯}{\varepsilon_{M}}$; (*ii*) Measured monopole fluid ergodicity $X\left(\omega ,T\right)=2k_{B}T\chi ”\left(\omega ,T\right)/\omega {\pi \upsilon \mu }_{0}S_{M}\left(\omega ,T\right)$; (*iii*) Measured frequency-dependent power law $b\left(T\right)$ of magnetization noise; (*iv*) Measured evolution of relative correlation length of dynamical heterogeneity. Evidently, all four characteristics of magnetic monopole dynamics span the same three ranges of temperature: thermally activated quasi-free monopole fluid (I) indicated in darker blue; the supercooled regime encompassing the monopole dynamical heterogeneity phenomenology (II) in white; and the yet unexplored regime (III) in light blue.

[13] $$\begin{array}{c} F_{\Phi }\left(\tau ,T\right)=N_{F}(T)\frac{1}{P-\tau }\sum_{t=0}^{P-\tau }\Phi_{p}\left(t,T\right)\;\Phi_{p}(t+\tau ,T), \end{array}$$

where $\begin{array}{c} N_{F}\left(T\right)=P/\left(\sum_{t=0}^{P}\Phi_{p}\left(t,T\right)\;\Phi_{p}\left(t,T\right)\right) \end{array}$ is the normalization constant ensuring $F\left(\tau =0,T\right)=1$. A 2D plot of $F_{\Phi }\left(\tau ,T\right)$ as determined using Eq. **13** is generated and then further interpolated in 10 mK temperature steps (*SI Appendix*, section VIII). The response function $\chi_{T}\left(\tau ,T\right)=\partial F_{\Phi }\left(\tau ,T\right)/\partial T$ is then calculated directly from that $F_{\Phi }\left(\tau ,T\right)$. Finally, the four-point dynamic susceptibility $\chi_{4}\left(\tau ,T\right)$ of the Dy_2_Ti_2_O_7_ monopole fluid is estimated from $\chi_{T}\left(\tau ,T\right)$ using Eq. **12** with specific heat data $c_{p}\left(T\right)$ as previously determined (47) (*SI Appendix*, section IX). Fig. 4*A* presents the resulting $\chi_{4}\left(\tau ,T\right)$ determined within the supercooled monopole fluid regime. It immediately reveals the increasing intensity in the evolution of the maxima in $\chi_{4}\left(\tau ,T\right)$ with falling temperature. These characteristics are patently consistent with long-established theory for $\chi_{4}\left(\tau ,T\right)$ in glass-forming molecular liquids (35–37) wherein, if dynamical heterogeneity is spatially compact, evolution of its length scale is then given by $\xi \left(T\right)\propto \sqrt[{3}]{{MAX(\chi }_{4}\left(\tau ,T\right))}$ (38–41, 48, 49). In consequence, Fig. 4*B* represents the measured temperature evolution of the maxima of $\chi_{4}\left(\tau ,T\right)$ and hence the evolving length scales of dynamical heterogeneity in a supercooled monopole fluid, with $\bar{\xi }(T)\equiv \xi (T)/\xi (T=1.5\;K)$ increasing by almost a factor of 8 across the supercooled regime. Furthermore, the time-evolution of the maxima in $\chi_{4}\left(\tau ,T\right)$ reveals a dramatic slowing of the dynamical heterogeneity. The time over which the dynamics retain maximum spatial correlation is the characteristic dynamical heterogeneity time $\tau_{4}$ (39). In theory, as the correlation length evolves approaching a glass transition, the relaxation time too must evolve as ever-larger regions of the material must rearrange cooperatively making such rearrangements exponentially rarer. For Dy_2_Ti_2_O_7_, the relaxation times $\tau_{4}\left(T\right)$ are determined by measuring the times at which $\chi_{4}\left(\tau ,T\right)$ is maximum for each temperature. In Fig. 4*C*, these times are compared to the relaxation times $\tau_{N}\left(T\right)$ derived from fitting the monopole noise spectrum to $S_{M}\left(\omega ,T\right)\propto \tau_{N}\left(T\right)/$$\left(1+{(\omega \tau_{N}\left(T\right)}^{b}\right)$, which themselves are known to be largely consistent with the relaxation times $\tau_{\chi }\left(T\right)$ from susceptibility measurements (29) (*SI Appendix*, section VII). All three independently determined relaxation times: $\tau_{4}\left(T\right),\tau_{N}\left(T\right)\;and\;\tau_{\chi }\left(T\right)$, are in good agreement for 0.5 K<T<1.5 K, thus revealing that the well-known VTF evolution of relaxation times $\begin{array}{c} \tau_{\chi }\left(T\right)\approx \tau_{N}\left(T\right)= \end{array}$$\begin{array}{c} A\exp(DT_{g}/(T-T_{g})) \end{array}$ in Dy_2_Ti_2_O_7_, is due to dynamical heterogeneity.

We amalgamate all the above results on the emerging phenomenology of dynamical heterogeneity in Dy_2_Ti_2_O_7_ spin-ice in Fig. 4*D*. Below T≈1500 mK, intense monopole current bursts emerge indicating large scale reorganizations of spin ice configurations. Their maximum magnitude relative to the generation/recombination monopole noise $R=max(\varepsilon_{B})/\overset{¯}{\varepsilon_{M}}$ grows rapidly, reaching maximum near T≈750 mK and eventually disappears near $T_{g}\approx 250\;mK$ (Fig. 4 *D, i*). This bimodality is due to elementary monopole generation recombination processes (32–34) falling into one class of monopole dynamics, with the monopole current bursts from dynamical reconfiguration of spin ice clusters being an intense second class (19, 21). Traversing this supercooled regime, a direct measure of monopole ergodicity $X\left(\omega ,T\right)$ diminishes cumulatively, reaching a minimum at $T≲T_{g}$ (Fig. 4 *D, ii*). Across the same regime the power law of magnetization noise collapses from the expected (19) value *b*=1.5 for quasi-free monopoles, toward *b*=1 approaching $T_{g}$ (Fig. 4 *D, iii*). The relative dynamical heterogeneity length scale $\bar{\xi }\left(T\right)$ increases significantly across the supercooled regime so that the volume of dynamically heterogeneous regions increases by a factor of approximately 500 as $T_{g}$ is approached (Fig. 4 *D, iv*). Overall this provides a far clearer and more comprehensive understanding of the evolution of spin ice dynamics from the thermally activated monopole plasma (state I), through the supercooled monopole fluid (state II) reaching the glass transition at $T_{g}\approx 250\;mK$, and into that ultralow temperature state (state III).

## Prospects

More generally, the striking correspondence between the phenomenology of dynamical heterogeneity of supercooled monopole fluids (Figs. 2–4) and that in supercooled glass forming liquids (2–8), emphasizes a potential universality among these ostensibly very distinct microscopic phenomena. In this regard, classical spin ice may provide a physically realized version of kinetically constrained models (KCM) for facilitated, heterogeneous relaxation during vitrification (50). But, unlike conventional KCMs where dynamical rules are postulates and their consequences then explored, the microscopic kinetic constraints of spin ice are directly identifiable. This is because, starting from a simple, clear, and quantitative Hamiltonian in Eq. **1**, the kinetic constraints on the spatiotemporal relaxation paths of spins emerge naturally from monopole motion through the dynamically evolving Dirac string network. The presence of such kinetic constraints in spin ice manifestly induces a slowing of spin relaxation, the subsequent appearance of dynamical heterogeneity as reported here, followed by an eventual glass state at $T_{g}\approx 250\;\mathrm{mK}$. Thus, in frustrated molecular glass-forming systems, the relaxation is plausibly controlled by rare, spatially correlated geometrical reconfigurations that become increasingly improbable with falling temperature while, in frustrated monopole glass-forming systems, the relaxation is controlled by rare, spatially correlated monopole reconfigurations of the 2-in:2-out spin arrangements, that become increasingly improbable with falling temperature. If correct, this represents a remarkable physics opportunity because spin ice provides a uniquely transparent realization of facilitated vitrification dynamics in which the kinetic constraints are microscopically well understood (17–22) and their spatiotemporal dynamical consequences are now directly accessible experimentally (Figs. 1–4).

## Supplementary Material

Appendix 01 (PDF)

Movie S1. Top: The evolution of the flux noise **Φ**_***p***_ (***t, T***) with falling temperature from ***T*** = 2500 **mK** to ***T*** = 15 **mK**. The flux noise signal, as it appears on screen, is converted to an audio signal and played over the video. Bottom: The simultaneous evolution of the monopole noise and monopole current burst energies at temperatures **15 mK** < ***T*** < 2500 **mK**.

## Data Availability

CSV data have been deposited in Zenodo (https://zenodo.org/records/20218561) (51).
