# Supplementary material for: Discovery of dynamical heterogeneity in a supercooled magnetic monopole fluid
Source: Proc Natl Acad Sci U S A. 2026 Jun 5;123(23):e2528457123. doi: 10.1073/pnas.2528457123 (PMC13250537; doi:10.1073/pnas.2528457123)
Supplement: Supplementary file 1 — Appendix 01 (PDF) [file pnas.2528457123.sapp.pdf]

PNAS

Supporting Information for
Discovery of Dynamical Heterogeneity in a Supercooled Magnetic Monopole Fluid.

Jahnatta Dasini, Chaia Carroll, Jack Murphy, Catherine Dawson, Hiroto Takahashi, Sudarshan Sharma, Fabian Jerzembeck, Stephen J. Blundell, Graeme Luke, J.C. Séamus Davis and Jonathan Ward

J.C. Séamus Davis and Jonathan Ward.
Email: jcseamusdavis@gmail.com and johnathanward@ucc.ie.

This PDF file includes:

Supporting text
Figures S1 to S14
Legends for Movies S1
SI References

Supporting Information Text

(I) Magnetic Monopole Dynamics in $Dy_2Ti_2O_7$

Spin Ice Monopoles. The paradigm of emergent magnetic monopoles in spin ice has been comprehensively successful over decades in explaining the experimentally observed dynamics and magnetic properties in dysprosium titanate (*52*). In such spin-ice compounds, e.g., $Dy_2Ti_2O_7$ and $Ho_2Ti_2O_7$, the lowest energy magnetic excitations are emergent magnetic charges (monopoles). Each Dy^{3+} or Ho^{3+} magnetic ion occupies a vertex of the corner-sharing tetrahedral sublattice and exhibits only two magnetic states with dipole moments $\mu \approx 10\ \mu_B$, pointing either towards or away from the center of each tetrahedron. Moreover, the lowest energy configuration of each tetrahedron is constrained by the dipolar-spin-ice Hamiltonian to have two spins pointing in and two pointing out (2-in:2-out), while the higher energy excitations are the effective magnetic charges ($+m$ for 1-in:3-out and $-m$ for 3-in:1-out) that are in some degree mobile. The magnetization dynamics (*53-60*) of both $Dy_2Ti_2O_7$ and $Ho_2Ti_2O_7$ are now widely viewed as due the correlated transport characteristics of emergent magnetic monopoles. On this basis the existence of a fluid of emergent magnetic monopoles is well attested. It is widely theorized that monopole dynamics continues even down to temperatures approaching $100\ \mathrm{mK}$ (*21,61-63*).

(II) Susceptibility and Relaxation Time Studies of $Dy_2Ti_2O_7$

The magnetic susceptibility $\chi(\omega,T) = \chi'(\omega,T) - i\chi''(\omega,T)$ of $Dy_2Ti_2O_7$ is known empirically with high precision *(59-61,64-71)*, as is the fact that below $T \approx 500\ \mathrm{mK}$ the linear-response relaxation rates in $Dy_2Ti_2O_7$ become ultra-slow *(57,72,73)*. Fig. S1 contains a review of measured linear-response relaxation times of $Dy_2Ti_2O_7$ using different experimental techniques with data from this work included.

A previous high precision study of the magnetic susceptibility of $Dy_2Ti_2O_7$ identified that the frequency-dependence of the magnetic susceptibility is very accurately parametrized by the Havriliak-Negami (HN) equation

$$\chi(\omega,T) = \chi_\infty + \chi_0(T)/\left(1+\left(i\omega\tau_\chi(T)\right)^{\alpha(T)}\right)^{\gamma(T)} \quad \text{(S1)}$$

Solving for the real and imaginary components of S1

$$\chi' = \chi_\infty + \chi_0 \frac{\cos(\gamma\varphi)}{\left(1+2(\omega\tau_\chi)^\alpha \cos\left(\frac{\pi\alpha}{2}\right)+(\omega\tau_\chi)^{2\alpha}\right)^{\gamma/2}} \quad \text{(S2)}$$

$$\chi'' = \chi_0 \frac{\sin(\gamma\varphi)}{\left(1+2(\omega\tau_\chi)^\alpha \cos\left(\frac{\pi\alpha}{2}\right)+(\omega\tau_\chi)^{2\alpha}\right)^{\gamma/2}} \quad \text{(S3)}$$

Here χ_∞ is the real value of χ in the $\omega \to \infty$ limit and τ_χ is the characteristic relaxation time. $\alpha(T)$ and $\gamma(T)$ describe the broadening and asymmetry of the distribution of relaxation times and

$$\varphi = \arctan(\left(\omega\tau_\chi\right)^\alpha \sin\left(\frac{\pi\alpha}{2}\right)/1 + \left(\omega\tau_\chi\right)^\alpha \cos\left(\frac{\pi\alpha}{2}\right)) \quad \text{(S4)}$$

The HN parametrization of $\chi(\omega,T)$ and the associated broad distribution of relaxation times, rather than a single relaxation time, implies a clear non-Debye character of the susceptibility. Indeed, the $\alpha(T)$ (broadening) and $\gamma(T)$ (asymmetry) terms describe the distribution of relaxation times and themselves evolve through the supercooled regime as previously discovered (*29*). Further, the linear-response relaxation times extracted by fitting directly measured $\chi(\omega,T)$ to S1 was demonstrated to be of the form

$$\tau_\chi(T) = A\exp\left(DT_g/(T-T_g)\right) \quad \text{(S5)}$$

where $A = 130 \pm 60\ \mu\mathrm{s}$ represents the quantum mechanical spin flip rate, $D = 13.6 \pm 5.0$ is the fragility index of the glass-forming state and $T_g \approx 240\ \mathrm{mK} \pm 30\ \mathrm{mK}$. This is the Vogel–Tammann–Fulcher (VTF) form characteristic of a supercooled glass-forming molecular liquids. In conventional supercooled liquids, $D < 5$ and $D > 50$ represent the fragile and strong extremes of glass formation respectively, while $\mathrm{Dy_2Ti_2O_7}$ rests in the intermediate. Hence, these forms for the susceptibility $\chi(\omega,T)$ and the relaxation time $\tau_\chi(T)$ indicate the existence of a supercooled monopole liquid in $Dy_2Ti_2O_7$, a deduction that is consistent with the empirical $\chi(\omega,T)$ and $\tau_\chi(T)$ reported by virtually all studies (Fig. S1).

(III) Combined Monopole Noise Spectrometer and AC Susceptometer

Design. Our monopole noise spectrometer assembly is shown schematically in Fig. S2. The sample holder is a hollow Macor cylinder onto which two persistent superconducting coils (signal pick up and field-cancellation coil) wound with opposite chirality are connected in-series with the input coil of the Quantum Design Model 550 SQUID. The SQUID couples a ~ *1* μH input coil into the ~ *100* pH SQUID coil, while maintaining a nominal critical current of ~ *10* μA. A cylindrical superconductive 'drive' coil for applying μT magnetic fields to the sample surrounds the pickup and astatic coils. The experiment is mounted at the mixing chamber plate of a dilution refrigerator. To expel and shield external magnetic fields, the SQUID is shielded within its own Niobium shield, this stage is surrounded by an additional outer Niobium cylindrical shield which is in turn enclosed in a larger cylindrical mu-metal shield. The spectrometer is mounted on the mixing chamber plate of a low-vibration cryogen-free dilution refrigerator that is vibrationally isolated and enclosed inside an acoustic isolation chamber. The refrigerator reaches a base temperature of $12\ \mathrm{mK}$.

Flux Noise Acquisition. The time-sequence of the magnetic flux generated by the sample $\Phi_p(t)$ is extracted using the inductances of the pickup coil L_p and input coil L_i, and $\mathcal{M}_i$ the mutual inductance to SQUID

$$\Phi_p(t,T) \equiv \Phi(t,T)/\left(\frac{\mathcal{M}_i}{2L_p+L_i}\right) \equiv \beta V(t,T)/G \tag{S6}$$

Where $\beta \equiv \left(\frac{\mathcal{M}_i}{2L_p+L_i}\right)^{-1} = 185.3$ derived from $L_p = 0.71\ \mu\mathrm{H}; L_i = 1.74\ \mu H; \mathcal{M}_i = 1.1 \times 10^{-8}\ \Phi_0/\mu A$ as set by the coil design. Using a SR560 Voltage Preamplifier, the signal is amplified and filtered by a low pass filter with a cutoff frequency f_{LP} of $3\ \mathrm{kHz}$, above which the SQUID is bandwidth limited. For temperatures above $600\ \mathrm{mK}$, an additional high pass filter is added with cutoff f_{HP} of $0.03\ \mathrm{Hz}$. The filtered SQUID output voltage V is recorded with 10 microsecond resolution for a total time of 1000 seconds.

Magnetic Susceptibility Data Acquisition. AC susceptibility measurements use a SR830 lock-in amplifier to measure the in-phase and out-of-phase components of the voltage output of the SQUID. An AC magnetic field $H(\omega)$ is synthesized by the lock-in amplifier by passing the Sine Output voltage signal ($10\ \mathrm{mV_{RMS}}$) through a $20\ \mathrm{k\Omega}$ resistor and RF filter before entering the solenoid drive coil (Fig. S2). The response of the $Dy_2Ti_2O_7$ sample is measured by the SQUID and fed into the lock-in amplifier. At each temperature setpoint, four frequency ranges are recorded: $0.1, 0.3, \ldots, 0.9\ \mathrm{Hz};\ 1, 2, \ldots, 10\ \mathrm{Hz};\ 11, 21, \ldots, 101\ \mathrm{Hz};\ 100, 200, 500, 1000$ and $2000\ \mathrm{Hz}$. The time constant is chosen to be $\tau_{LI} \geq 3(1/f_{\min})$ for the respective frequency ranges. The sensitivity of the lock-in amplifier is set to $20\ \mathrm{mV/nA}$ for $T < 600\ \mathrm{mK}$ and $50\ \mathrm{mV/nA}$ for $T \geq 600\ \mathrm{mK}$.

(IV) Monopole Noise and AC Susceptibility Analysis

Noise Analysis. The magnetization is related to the output voltage of the SQUID as

$$V(t,T) = \Phi_p(t,T)\ G/\beta = \frac{M(t,T)}{C_0} \tag{S7}$$

where $C_0 \equiv \left(\frac{\Phi_0}{\beta NAF}\right) = 2.1 \times 10^{-9}\ \mathrm{JT^{-1}V^{-1}m^{-3}}$ is calibrated accurately for our experimental geometry. The time-sequences of magnetization fluctuations are recorded from $V(t)$ for each temperature *T*. The power spectral density of magnetization noise $S_M(\omega,T)$ is derived using

$$S_M(\omega,T) \equiv \lim_{\mathcal{T}\to\infty} \frac{1}{\pi\mathcal{T}} \left| \int_{-\frac{\mathcal{T}}{2}}^{\frac{\mathcal{T}}{2}} M(t) e^{-i\omega t} dt \right|^2 \tag{S8}$$

The complete frequency and temperature dependence of the magnetization noise spectral density is shown in Fig. S3.

Magnetic Susceptibility Analysis. To calculate the AC Susceptibility, it is convenient to first define a pre-factor $F_1 = C_\chi(2L_p + L_i)/\mathcal{M}_i$ for converting the SQUID output voltage to magnetic flux in the pickup coil. C_χ = 0.0073 V/Φ_0 is a value intrinsic to the SQUID electronics, while $L_p = 0.71\ \mu\mathrm{H}$ and input coil $L_i = 1.74\ \mu\mathrm{H}$ represent the inductances of the pickup coil and input coil respectively. $\mathcal{M}_i = 1.1 \times 10^{-8}\ \Phi_0/\mu\mathrm{A}$ represents the mutual inductance of the SQUID circuitry (Fig. S2). To convert flux to *B*-field, a second pre-factor $F_2 = \Phi_0/N_{coil}A_{coil}F$ is used. $N_{coil} = 16$ is the total number of turns in

the pickup coil, $A_{coil} = 3.843 \times 10^{-6}\ \mathrm{m}^2$ is the pickup coil cross-sectional area, $F = 0.57$ is the filling factor. At each frequency 10 in-phase (X) and out-of-phase (Y) voltage values are collected from the Lock-In, from which average values V_x and V_y are calculated. Quantitatively accurate real and imaginary magnetic susceptibilities are then found using

$$\chi'(\omega, T) = \frac{V_x(\omega,T)}{\mu_0 H_{mod}}\left(\frac{1}{F_1 F_2}\right) \tag{S9}$$

$$\chi''(\omega, T) = \frac{-V_y(\omega,T)}{\mu_0 H_{mod}}\left(\frac{1}{F_1 F_2}\right) \tag{S10}$$

χ' and χ'' are fitted to the HN equations S2 and S3 respectively and presented on both a linear and logarithmic scale in Fig. S4.

(V) Ergodicity from Fluctuation-Dissipation Theorem Analysis

Examining Ergodicity of the Monopole Fluid. If the Fluctuation-Dissipation Theorem (FDT) is obeyed for $Dy_2Ti_2O_7$, the magnetization noise $S_M(\omega, T)$ would be directly related to the imaginary AC susceptibility χ'' by

$$S_M(\omega, T) = \frac{2k_B T}{\omega\pi\upsilon\mu_0}\chi''(\omega, T) \tag{S11}$$

wherein SI units are used throughout so that $\chi''(\omega, T)$ is unitless. Using measured $S_M(\omega, T)$ and $\chi''(\omega, T)$, the left-hand side of S11 is plotted against the right-hand side for frequencies in the range $0.3 - 2000$ Hz (Fig. S5). To improve the reliability of the low temperature noise data where the signal to noise ratio is lowest, each $S_M(\omega, T)$ (which has the contribution from the empty coil subtracted) curve is averaged over 20 second segments, meaning the error bars (~1%) are not overlapping with the noise floor even for the lowest magnitude noise data. Each temperature, differentiated by color in Fig. 1F in the main text, contains several points on the curve corresponding to the frequencies used in the experiment. To quantify the validity of the FDT, a ratio $X(\omega, T)$ is defined as

$$X(\omega, T) = \frac{2k_B T}{\omega\pi\upsilon\mu_0}\frac{\chi''(\omega,T)}{S_M(\omega,T)} \tag{S12}$$

Where $X \approx 1$, the FDT is obeyed while $X < 1$ indicates a violation of FDT due to a loss of ergodicity of the system and the presence of excess noise. To show the temperature evolution, $\overline{X}$(T) is defined to be $X(\omega, T)$ averaged over all experimental frequencies. $\overline{X}$(T) is shown in Fig. 4Dii in the main text.

(VI) Analysis of Time-Resolved Monopole Noise

Flux at Pickup Coil from SQUID Output. The SQUID output voltage signal $V(t, T)$ is recorded with $10\ \mu\mathrm{s}$ precision. $V(t, T)$ is calibrated by the design of the circuit (Fig. S2) to accurately measure the flux produced by the $Dy_2Ti_2O_7$ crystal as it threads the pickup coil $\Phi_p(t, T)$ as in Eqn. S7. A typical $\Phi_p(t, T)$ signal is shown in Fig. 1B in the main text.

Magnetic Monopole Current. The monopole current $J(t, T)$ is in principle related to the flux $\Phi_p(t, T)$ by

$$J(t, T) \equiv \dot{\Phi}_p(t, T)/\mu_0 \tag{S13}$$

When calculating the time derivative of a noisy $\phi_p(t, T)$ signal, an $80\ \mu\mathrm{s}$ boxcar average is first applied to suppress artifacts that may arise from numerical differentiation. The derivative $\dot{\Phi}_p(t, T)$ is calculated using the Central Difference Method:

$$\dot{\Phi}_p(t, T) = \frac{\Phi_p(t+\Delta t, T) - \Phi_p(t-\Delta t, T)}{2\Delta t} \tag{S14}$$

Using S13 the current $J(t, T)$ is calculated. In this analysis, only the magnitude of current noise $|J(t, T)|$ as no net current is observed. In particular, the distribution of occurrence rate $r_{|J|}$, is calculated by considering the number $\eta(|J|)$ of times a given current magnitude $|J|$ occurs in a fixed time interval *P*: $r_{|J|} = \eta(|J|)/P$. Further analysis examines the mean of monopole current magnitudes $|J|$ versus temperature T. Results of the magnitude of monopole current $|J(t, T)|$ are presented in Fig. 2 in the main text. Two types of monopole current occur within this current distribution: rearranging S13 the relation which directly relates $J(t)$ to changes in the flux $\Phi_p(t)$ is

$$\mu_0 \int_{t_i}^{t_f} J(t', T)\, dt' = \Phi_p(t_f, T) - \Phi_p(t_i, T) \tag{S15}$$

This means that intense current bursts existing over extended time periods produce excursions in $\Phi_p(t,T)$ far larger than those generated by conventional monopole noise. This effect is seen directly in histograms of $|\Phi_p(t,T)|$ as show in Fig. S6.

Energetics: Continuous Distribution of Energies. To understand the energy scales of the monopole phenomena, the relation

$$\varepsilon(t,T) \equiv \Phi_p^2(t,T)/2L_p \tag{S16}$$

is used. In general, a flux change Φ in a persistent superconducting coil of inductance L produces a supercurrent given by $I = \Phi/L$: the coil then stores energy $\varepsilon = \Phi^2/2L$. Hence in our studies, where the only source of energy is the magnetization dynamics of the sample, the flux produced by the $Dy_2Ti_2O_7$ sample at the pickup coil, Φ_p, represents a monopole current event with energy $\varepsilon_p = \Phi_p^2/2L_p$. Fig. 3A in the main text shows that monopole current bursts, which are large collective increases in the flux always followed by a collective reversal, typically occur on timescales of order ~ 1 ms. The square of the flux noise signal Φ_p^2 is averaged in an 80 μs window for consistency with the current analysis. The continuous $\Phi_p^2(t,T)$ signal (Fig. S7) is converted to energy using S16. For reference, the noise picked up purely by the circuitry (no $Dy_2Ti_2O_7$ sample) is shown in black in Fig. S7. The distribution of the occurrence rate $r(\varepsilon,T)$ of events with energy ε is calculated by considering the number $m(\varepsilon)$ of times a given energy ε occurs in the continuous energy signal within a fixed time interval *P*: $r(\varepsilon) = m(\varepsilon)/P$. The striking emergence (see Movie and Audio S1) of a second gaussian distribution in the range $250\ \text{mK} \lesssim T \lesssim 1500\ \text{mK}$, corresponding to the emergence of current bursts in the Φ_p^2 signal, prompts further analysis. To do so, a given $r(\varepsilon,T)$ distribution is fit to a bi-modal model, where the overall distribution is represented by the sum of two unique gaussian functions

$$\varepsilon_M + \varepsilon_B = A_M \exp\left(-\frac{(\varepsilon-\overline{\varepsilon_M})^2}{2\sigma_M^2}\right) + A_B \exp\left(-\frac{(\varepsilon-\overline{\varepsilon_B})^2}{2\sigma_B^2}\right) \tag{S17}$$

Here subscript M denotes the noise produced by conventional monopole noise and subscript B denotes the noise produced by transient bursts of monopole current. In the cases where this model fails (i.e., one of the distributions goes to zero, or the two gaussians are almost completely overlapping), it implies that the current bursts are no longer present in the signal. Results of the analysis of the continuous distribution of energies are presented in Fig. 3 in the main text.

(VII) Monopole Noise Power Law.

The magnetization noise floor, as measured using an empty pickup coil, is subtracted from the measured $Dy_2Ti_2O_7$ magnetization noise at each temperature. The resulting magnetization noise spectrum S_M reveals the true contribution to the magnetization signal from the monopoles. S_M is fitted using a least-squares method to the standard equation

$$S_M(\omega,T) = \frac{\sigma_M^2(T)\tau_N(T)}{\left(1+(\omega\tau_N(T))^{b(T)}\right)} \tag{S18}$$

in the frequency range $0.05 - 10{,}000\ \text{rad/s}$. For optimal fitting, only data two times greater than the noise floor are included in the fit. The power law exponent $b(T)$, relaxation time $\tau_N(T)$ and magnetization variance $\sigma_M^2(T)$ are free parameters of the fit. Within our 1000s acquisition, it is possible to access very slow relaxation times $\tau_N(T)$ on the order of tens of seconds (Fig. S1). The quality of fit is indicated by the inset of Fig. S8. Fig. 4Diii in the main text shows the temperature dependence of the monopole noise power law $b(T)$; a sharp decrease from the predicted $b = 1.5$ towards $b = 1$ is seen in the $T \to 0$ limit.

(VIII) Autocorrelation Function

The autocorrelation function $F_\Phi(\tau,T)$ is defined $F_\Phi(\tau,T) \equiv N_F(T)\langle\Phi_p(t,T)\Phi_p(t+\tau,T)\rangle_t$. $F_\Phi(\tau,T)$ is calculated from the discrete flux signal $\Phi_p(t,T)$, in the interval $P = 1000$ s, by:

$$F_\Phi(\tau,T) = N_F(T)\frac{1}{P-\tau}\sum_{t=0}^{P-\tau}\Phi_p(t,T)\Phi_p(t+\tau,T) \tag{S19}$$

where $N_F = P/(\sum_{t=0}^{L}\Phi_p(t,T)\Phi_p(t,T))$ normalizes $F_\Phi(\tau,T)$ ensuring $F_\Phi(\tau=0) = 1$. $F_\Phi(\tau,T)$ is calculated from each $\Phi_p(t,T)$ and interpolated (linear) at $\Delta_T = 10$ mK intervals to create a smooth

surface. The evolution of $F_\Phi(\tau,T)$ from the monopole fluid regime to the deeply supercooled regime is shown in Fig. S9. The evolution of the relaxation time is clearly identified and correlated monopole motion increases dramatically as temperature falls.

(IX) Four-Point Dynamical Susceptibility: Theory and Experiment

Deriving Dynamical Susceptibility from the Autocorrelation Function. Numerical works on supercooled glass-forming liquids show the emergence of spatially heterogeneous dynamics *(74)* upon cooling into the supercooled phase. Subsequent studies have attempted to quantify this fact by probing four-point dynamic susceptibility

$$\chi_4(\tau,T) \equiv N\langle \delta C^2(t,\tau,T)\rangle_t \quad \text{(S20)}$$

which measures the spatiotemporal correlations of fluctuations about the average. $C_A(t,\tau,T) = A(t,T)A(t+\tau,T)$ represents the two-point correlation of an instantaneous fluctuation of a mean-zero local observable $A(\vec{r},t,T)$. Here $\langle \delta C^2\rangle = \langle C_A^2\rangle - \langle C_A\rangle^2$ is the ensemble-averaged fluctuation of $C_A^2(t,\tau,T)$ about its average, and N is the number of particles. The normalized correlation function $F_A(\tau,T)$ is equivalent to the average of $C_A(t,\tau,T)$ over the time t of the observation: $F_A(\tau,T) \equiv \langle C_A(t,\tau,T)\rangle_t / \langle C_A(t,0,T)\rangle_t$.

As discussed in the main text, this spatiotemporal information is not currently available for molecular glass forming liquids. Alternatively, using an approach based on fluctuation-dissipation theorem, the dynamic susceptibility can be defined from the time-dependent correlator $F(\tau,T)$. First, the response function $\chi_T(\tau,T)$ is defined as the response of $F(\tau,T)$ to temperature variations

$$\chi_T(\tau,T) = \frac{\partial F(\tau,T)}{\partial T} \quad \text{(S21)}$$

This equation also holds in the frequency domain $\chi_T(\omega,T) = \partial\tilde{F}(\omega)/\partial T$ where $\tilde{F}(\omega)$ can be the dielectric susceptibility. In a molecular liquid, the fluctuation-dissipation relation

$$k_B T^2 \chi_T(\tau,T) = N\langle \delta C(t,\tau,T)\delta H(t,0,T)\rangle \quad \text{(S22)}$$

can be established, where k_B is the Boltzmann constant, $\delta H(t,0,T)$ the fluctuating enthalpy per particle and $\delta C(t,\tau,T)$ is the instantaneous value of the correlation function $F(\tau,T)$. Importantly here, $C(t,\tau,T)$ and $H(t,\tau,T)$ are sums over local contributions (*75*)

$$C(t,T) = \frac{1}{V}\int d^3\vec{r}\; c(\vec{r},t,T) \quad \text{(S23)}$$

$$H(t,\tau,T) = \frac{\sqrt{k_B c_p(T)}T}{V}\int d^3\vec{r}\; h(\vec{r},t,T) \quad \text{(S24)}$$

where V is the volume of the sample. From thermodynamics, the specific heat at constant pressure $c_p(T)$ here sets the scale of the enthalpy fluctuations $\langle \delta H(t,0,T)^2\rangle_t = Nk_B c_p(T)T^2$. Using translational invariance, Eqn. S22 is rewritten as

$$\sqrt{\frac{k_B}{c_p(T)}}T\chi_T(\tau,T) = \int d^3\vec{r}\;\langle \delta c(\vec{r},t,\tau,T)\delta h(0,t,0,T)\rangle_t \quad \text{(S25)}$$

where $\rho = N/V$ is the density. In the same way the four-point correlation function is the variance of the two-point correlation function $\langle \delta C^2(t,\tau,T)\rangle_t \propto \int d^3\vec{r}\,\langle \delta c(\vec{r},t,\tau,T)\delta c(r,t,0,T)\rangle_t$, the space integral of the three-point correlation is the covariance of the dynamic correlation with energy fluctuations: $\langle \delta C(t,\tau,T)\delta H(t,0,T)\rangle_t \propto \int d^3\vec{r}\;\langle \delta c(\vec{r},t,\tau,T)\delta h(0,t,0,T)\rangle_t$. This powerful relationship shows that $\chi_T(\tau,T)$, and therefore the flux signal $\Phi_p(t,T)$ itself, allows one to directly probe the spatial correlations between local fluctuations of the dynamics and that of the enthalpy.

It is important to note here that the flux is a valid choice of measurable quantity to observe these effects despite having no spatial coordinate. Using a volume averaged quantity like Φ_p (directly proportional to magnetization $M(t)$) to calculate $C(t,\tau,T)$ represents approximately taking the $q = 0$ Fourier component of the integral $C(t,\tau,T) = \frac{1}{V}\int d^3\vec{r}\; c(\vec{r},t,\tau,T) = \frac{1}{V}\int d^3\vec{r}\;\Phi(\vec{r},t+\tau,T)\;\Phi(\vec{r},t,T) = \frac{1}{V(2\pi)^3}\int d^3\vec{q}\;\Phi(\vec{q},t+\tau,T)\;\Phi(-\vec{q},t,T)$. In this sense, the spatial correlation is not directly in the measurable quantity but can be inferred through the relationship between $\chi_T(\tau,T)$ and $\chi_4(\tau,T)$. This approach has been successfully demonstrated in experiments on colloidal hard spheres and glass-forming glycerol.

Specifically, χ_T can be related to χ_4 using the Cauchy-Schwartz inequality:

$$\langle \delta H(t,0,T)\delta C(t,\tau,T)\rangle_t^{\,2} \leq \langle \delta H(t,0,T)^2\rangle_t \langle \delta C(t,\tau,T)^2\rangle_t \quad \text{(S26)}$$

Using Eqn. S22 to substitute for $\chi_T(\tau,T)$ on the left-hand side and Eqn. S20 to substitute for $\chi_4(\tau,T)$ on the right-hand side

$$\frac{1}{N^2}(k_B T^2)^2[\chi_T(\tau,T)]^2 \leq \frac{1}{N}(k_B c_p(T) T^2)\frac{1}{N}\chi_4(\tau,T) \quad \text{(S27)}$$

$$\chi_4(\tau,T) \geq \frac{k_B T^2}{c_p(T)}[\chi_T(\tau,T)]^2 \quad \text{(S28)}$$

Now, the experimentally accessible $\chi_T(\tau,T)$ can be used to tightly bound the dynamic susceptibility $\chi_4(\tau,T)$. In general, to equate the two, all fluctuating quantities Δ_i in the system should be considered (*76*). $\chi_4(\tau,T)$ is the sum of all contributions from fluctuations: $\chi_4(\tau,T) = \sum k_i(T)\, \chi_{\Delta_i}(\tau,T)$. Here $k_i(T)$ represents the collection of pre-factors relevant to each fluctuation. To first order, the temperature and monopole density ρ_{mono} fluctuations are relevant in the supercooled monopole fluid of $Dy_2Ti_2O_7$,

$$\chi_4(\tau,T) \approx \frac{k_B T^2}{c_p(T)}[\chi_T(\tau,T)]^2 + \rho k_B T \kappa_T \rho_{\text{mono}}^2 \left[\chi_{\rho_{mono}}(\tau,T)\right]^2 \quad \text{(S29)}$$

where κ_T is the isothermal compressibility and ρ the density of $Dy_2Ti_2O_7$. Studies on fragile liquids (*77*), which is the case of the monopole fluid in $Dy_2Ti_2O_7$, reveal that the density term is negligible, therefore χ_T itself provides a good approximation of χ_4

$$\chi_4(\tau,T) \approx \frac{k_b T^2}{c_p(T)}[\chi_T(\tau,T)]^2 \quad \text{(S30)}$$

Because the FDT begins to be violated in the supercooled monopole fluid at $T \approx 500$ mK, $\chi_4(\tau,T)$ is only considered above this temperature.

Response Function Analysis of Supercooled Monopole Fluids. The dynamical susceptibility described above has been derived, simulated, and experimentally measured for conventional molecular glass forming liquids. However, this approach need not be restricted to conventional glass formers but may also be relevant to spin glasses. It has been shown that the growth of the dynamical susceptibility derived from Eqn. S30 reveals unique dynamical length scales that are independent of the choice of dynamics and, most importantly, independent of the choice of ensemble. The requirements to apply the above concepts are the validity of the fluctuation-dissipation theorem and that the system must obey the general thermodynamic relation that the temperature derivative of a global dynamical correlator is related to cross-correlations (via fluctuation–dissipation) between local dynamical fluctuations and energy (enthalpy). The monopole fluid can be well interpreted within the grand canonical ensemble (*78*) and remains ergodic at $T \geq 500$ mK, hence the dynamical susceptibility of the monopole fluid may be described as above.

The use of flux noise $\Phi(t)$ measurements allows greatly increased sensitivity at lowest frequencies because, when FDT holds, $S_M(\omega,T)/\chi''(\omega,T) \propto T/\omega$, so that in terms of its signal/noise ratio, accessing extremely slow $\omega \to 0$ dynamics using AC susceptibility $\chi''(\omega,T)$ is enormously less efficient than doing so from long-time magnetization noise $\Phi(t)$ measurements.

Calculating the Dynamical Susceptibility from Noise Data. The multipoint correlation function $\chi_T(\tau,T)$ is calculated directly from the $F_\Phi(\tau,T)$ surface shown in Fig. S9 using the Central Difference Method

$$\chi_T(\tau,T_i) \equiv \frac{\partial F_\Phi(\tau,T_i)}{\partial T} = \frac{F_\Phi(\tau,T_{i+1}) - F_\Phi(\tau,T_{i-1})}{2\Delta_T} \quad \text{(S31)}$$

$\chi_T(\tau,T)$ is shown across the supercooled regime in Fig. S10. The heat capacity at constant pressure $c_p(T)$ is calculated from the data reported in (*47* pp 5). Using $c_p(T)$ and $\chi_T(\tau,T)$ data as shown in Fig. S10, $\chi_4(\tau,T)$ is calculated using Eqn. S30. Because the low-frequency monopole noise grows exponentially with falling temperature, probing the dynamic susceptibility using noise allows for far greater signal-to-noise ratio than ac susceptibility experiments would allow for.

(X) Nonlinear Susceptibility Studies of Dynamical Heterogeneity

Growing Length Scales in Amorphous Materials. In spin glasses, it has been shown (*79*) that amorphous order can be detected only via higher order magnetic susceptibilities, because the four-point susceptibility $\chi_4(t)$ and the growing cooperative volume ,while inaccessible experimentally, are shown theoretically to be connected to the higher order susceptibility (*13*). Higher order susceptibilities have been measured directly in spin glasses and have been extracted indirectly in supercooled liquids. In spin glasses, higher order susceptibilities have been measured directly by non-linear magnetization (*79*). In supercooled liquids, higher order susceptibilities have been extracted from the temperature derivative (as derived in Section IX above) of the linear

susceptibility (*45, 80-82*). In all of these models, the non-linear susceptibilities of amorphous materials reveal growing length scales approaching the freezing/glass transition temperature. Beyond susceptibility, the growing length scale associated with dynamical heterogeneity has also been measured in a polymer using multidimensional NMR techniques (*83*).

(XI) Dy2Ti2O7 Samples

Sample Growth. The single crystal rod-shaped $Dy_2Ti_2O_7$ samples (Fig. S11) are grown by floating zone method. High purity (99.99%) Dy_2O_3, and TiO_2 are mixed and heated to 1400 °C for 40 hours. The mixture is ground immediately, then heated for 12 hours. The resulting powder is packed into a rod, then sintered at 1400° C for 12 hours. A long piece of the sintered rod is used as a feed rod while a small piece is used as the seed. The crystals are grown in $0.4\ \mathrm{MPa}$ oxygen pressure at 4 mm/hour using a two-mirror NEC furnace where the feed and seed rods are counter-rotated at $30\ \mathrm{rpm}$.

Sample Demagnetization. The demagnetization factor for a rod-shaped sample with the field applied along the length of the rod is zero for an infinitely long sample. However, real experiments have finite length and therefore a finite demagnetization factor needs to be accounted for. A finite cuboid has a demagnetizing field B_d of

$$\vec{B}_d = -\boldsymbol{N}(\vec{r})\mu_0\vec{M} \qquad \text{(S32)}$$

where $\boldsymbol{N}(\vec{r})$ is the position dependent demagnetization factor. For a cuboid with dimensions $2a \times 2b \times 2c$, $N(\vec{r})$ is given by *(84)*

$$\boldsymbol{N}_{ii}(\vec{r}) = \frac{1}{4\pi}\sum_{\alpha=\pm1}\sum_{\beta=\pm1}\sum_{\gamma=\pm1}\tan^{-1}(f_i(\alpha x, \beta y, \gamma z)) \qquad \text{(S33)}$$

With $i = x, y, z$ and the functions f_i are given by

$$f_x(x,y,z) = \frac{(b-y)((c-z)}{(a-x)\sqrt{[(a-x)^2+(b-y)^2+(c-z)^2]}} \qquad \text{(S34)}$$

$$f_y(x,y,z) = \frac{(a-x)((c-z)}{(b-y)\sqrt{[(a-x)^2+(b-y)^2+(c-z)^2]}} \qquad \text{(S35)}$$

$$f_z(x,y,z) = \frac{(b-y)((a-x)}{(c-z)\sqrt{[(a-x)^2+(b-y)^2+(c-z)^2]}} \qquad \text{(S36)}$$

Considering the superconducting pickup coil is wound around the centre of the DTO sample, the greatest contribution to the demagnetizing field will be along the z axis at the centre of the coil, $\boldsymbol{N}_{zz}(0,0,0)$. Using the known geometry of the sample $2a = 0.98\ \mathrm{mm}, 2b = 1.31\ \mathrm{mm}, 2c = 6.23\ \mathrm{mm}$, this gives an approximate demagnetization factor of

$$\boldsymbol{N}_{zz(0,0,0)} \sim 0.01 \qquad \text{(S37)}$$

which was part of the experimental design concept. Therefore, the estimated demagnetization factor is roughly 1%, which is insignificant to observations reported in this work.

Stuffed Defects. The floating zone method used here for growing pyrochlore titanate crystals typically produces clean crystals with ~1% stuffing fraction *(85)*. These stuffed sites undoubtedly produce their own dynamics which have not been extensively studied in previous works, while certainly influencing the monopolar dynamics particularly at low temperatures (*86*). At 1.5 K when the current bursts begin to emerge, the monopolar density is at least an order of magnitude greater than the defect density. Further, at this temperature the magnetization noise spectrum is consistent with a fluid of magnetic monopoles as the source of magnetization intrinsic to the sample. It is therefore implausible that the monopole bursts can be attributed to site disorder/stuffing in the crystal. A key future project could be the investigation of the effects of stuffed defect density on the supercooled monopole fluid state of DTO.

Stuffed Defects. This sequence of experiments was repeated with three different $Dy_2Ti_2O_7$ samples. Within typical margins due to geometrical effects, all samples produced equivalent phenomenologies (Fig. S12). The reported magnetization noise and susceptibility data show excellent agreement with previously reported data on the material, validating the quality of samples and experimental assembly.

Figures

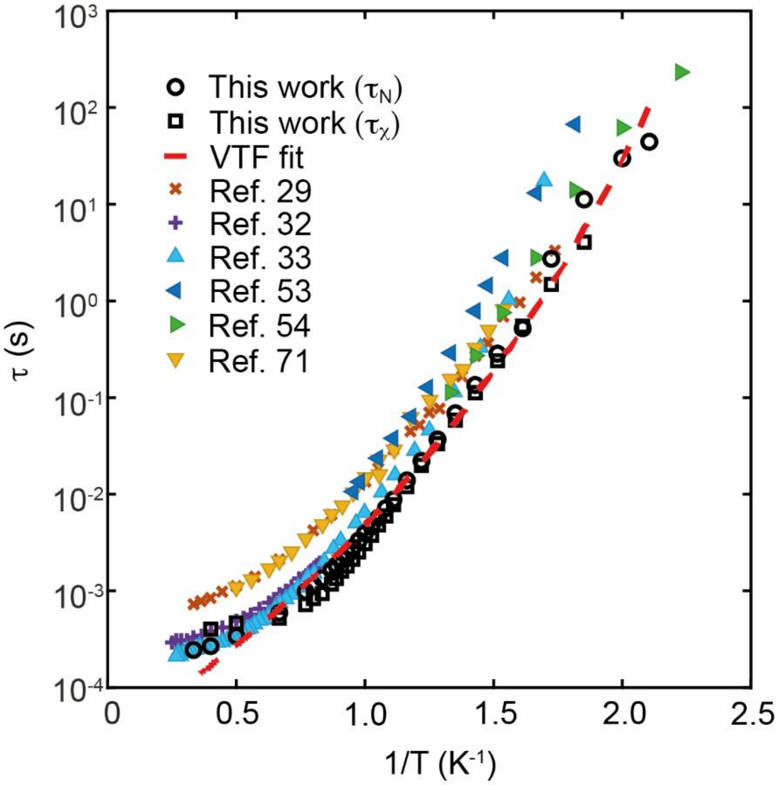

Fig. S1. The linear-response relaxation time $\boldsymbol{\tau}$ measured by fitting our magnetization noise $\boldsymbol{S_M}$ (black circles) and AC susceptibility $\boldsymbol{\chi}$'' (black squares) is compared to related measurements in the literature (coloured symbols) and found to be consistent with previously reported values. Below $\boldsymbol{T \approx 500\ \mathrm{mK}}$, $\boldsymbol{\tau}$ becomes inaccessible to linear-response experiments due to its divergent behavior approaching $\boldsymbol{T_g = 240\ \pm 30\ \mathrm{mK}}$. Refs. 32 & 33 derive $\boldsymbol{\tau}$ from noise measurements, while Refs. 29, 53, 54 and 71 derive $\boldsymbol{\tau}$ from AC susceptibility measurements. Deviations in values of $\boldsymbol{\tau}(\boldsymbol{s})$ between the referenced data sets are attributed to due to different sample geometries and experimental configurations. However, the VTF form of relaxation time is held throughout.

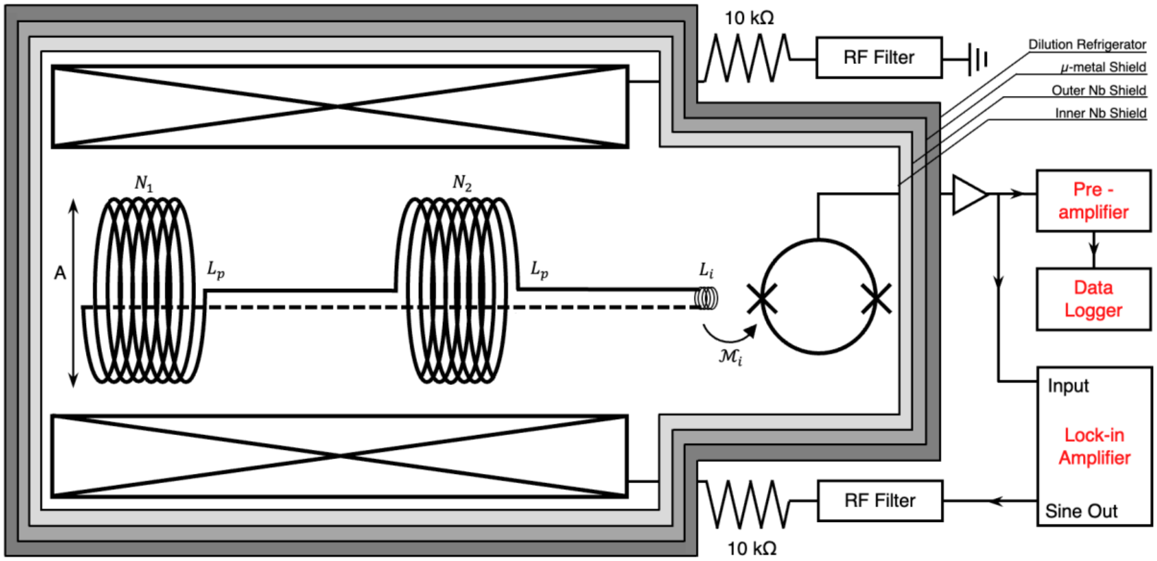

Fig. S2. The schematic of our combined monopole noise spectrometer and AC susceptometer. The circuit diagram illustrates the simultaneous monopole flux-noise and AC susceptibility measurement.

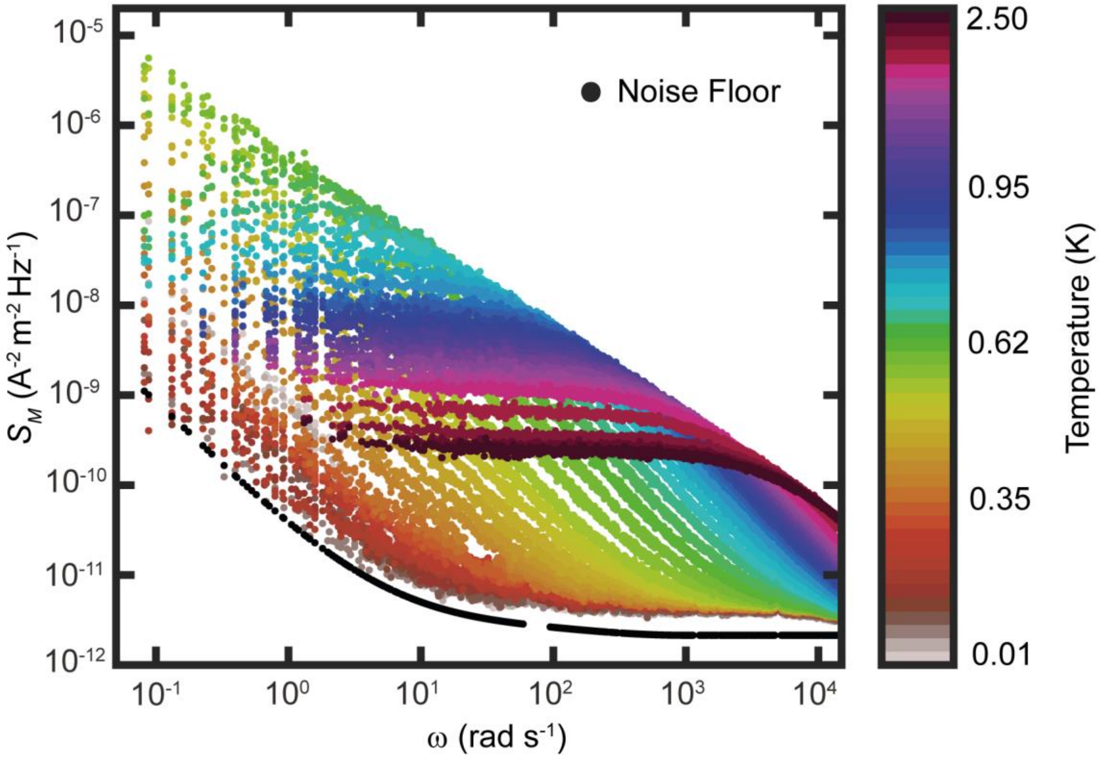

Fig. S3. Unprocessed magnetization noise power spectral density data $\boldsymbol{S_M(\omega, T)}$ versus $\boldsymbol{T}$. The measured empty-coil noise floor is plotted as a black curve and lies below the monopole noise spectra.

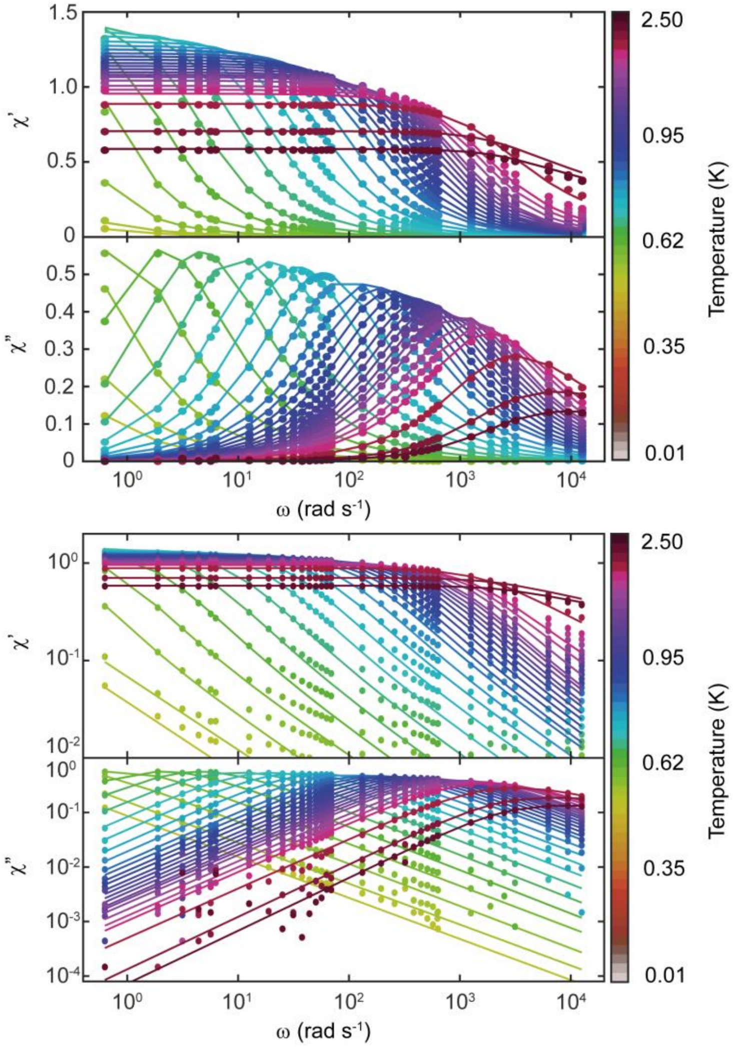

Fig. S4. T The real component (top) of the magnetic AC susceptibility $\chi'(\boldsymbol{\omega}, \boldsymbol{T})$ is fitted to its parametric equation S2. Below $\mathbf{500\ mK}$ the fit fails ($\boldsymbol{R}^{2} < \mathbf{0.99}$). The imaginary component (bottom) of the magnetic AC susceptibility $\chi''(\boldsymbol{\omega}, \boldsymbol{T})$ is fitted to its parametric equation S3. The evolution of the monopole linear-response relaxation time is reflected clearly by the shift of the peak in $\chi''(\boldsymbol{\omega}, \boldsymbol{T})$ towards lower frequencies as the temperature is decreased. Below $\mathbf{500\ mK}$, where the peak is no longer in our experimental window, the fit fails ($\boldsymbol{R}^{2} < \mathbf{0.99}$). Data that cannot be parametrized by S3 are included in Fig. S5. The lower figure shows the identical susceptibility data and corresponding fits presented on a log-log scale.

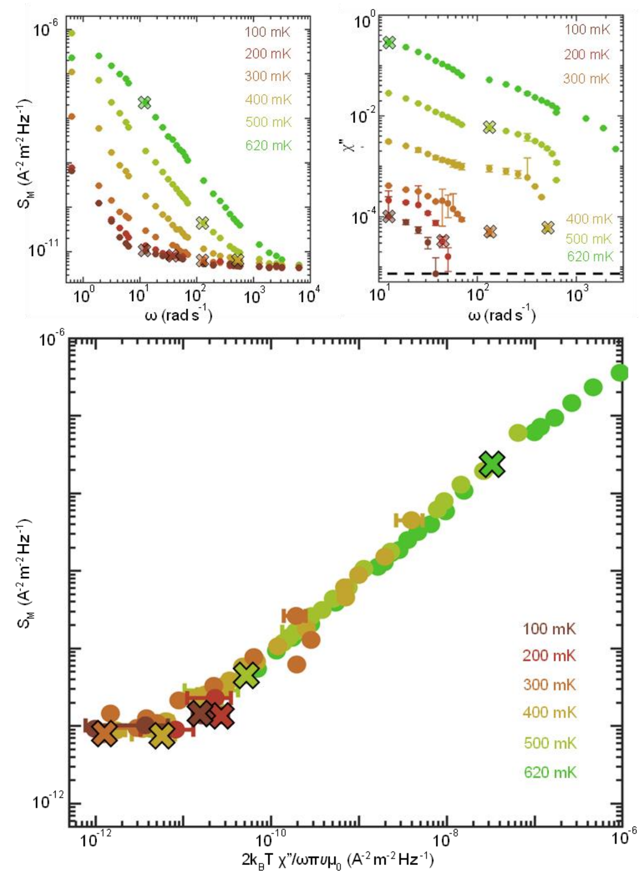

Fig. S5. Magnetization noise (top left) S_M at 100 mK, 200 mK, 300 mK, 400 mK, 500 mK, and 620 mK. Each curve shows magnetization noise data at the corresponding frequencies to the susceptibility measurements in the next panel. The error in the noise is less than 1% of the signal in all cases, so the error bars are not included beyond this panel. Imaginary susceptibility (top right) χ" at the same temperatures as the top-left panel. The experimental noise floor is plotted at the base of the figure. Bottom: The left-hand side of the fluctuation-dissipation relation S11 (Y) is compared against the right-hand side (X). One data point at each temperature is represented by an 'X' as a guide to the eye. The same points are highlighted in the top panels to identify the pair of unprocessed noise and susceptibility values yielding that data point. At temperatures below 300 mK, a violation of the fluctuation-dissipation theorem is observed, as the linear relationship between the simultaneously measured magnetization noise and imaginary susceptibility fails.

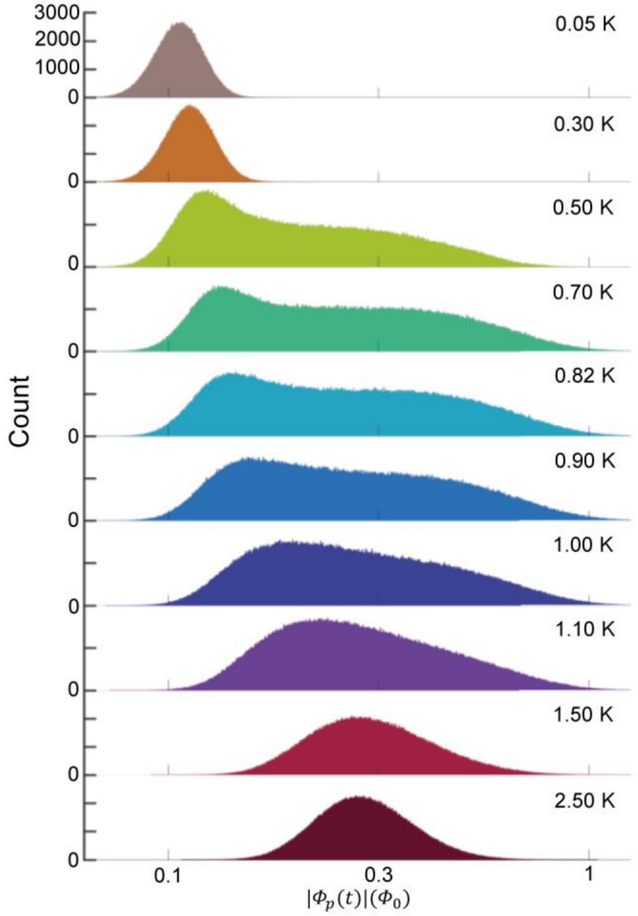

Fig. S6. Typical histograms of $|\boldsymbol{\Phi_p(t)}|$. Conventional monopole current with a single Gaussian distribution persists until $T \approx 1500$ mK. A second current source, due to intense monopole current bursts appears below this temperature resulting in a bimodal distribution of probabilities. Below $T \lesssim 250$ mK the current bursts disappear.

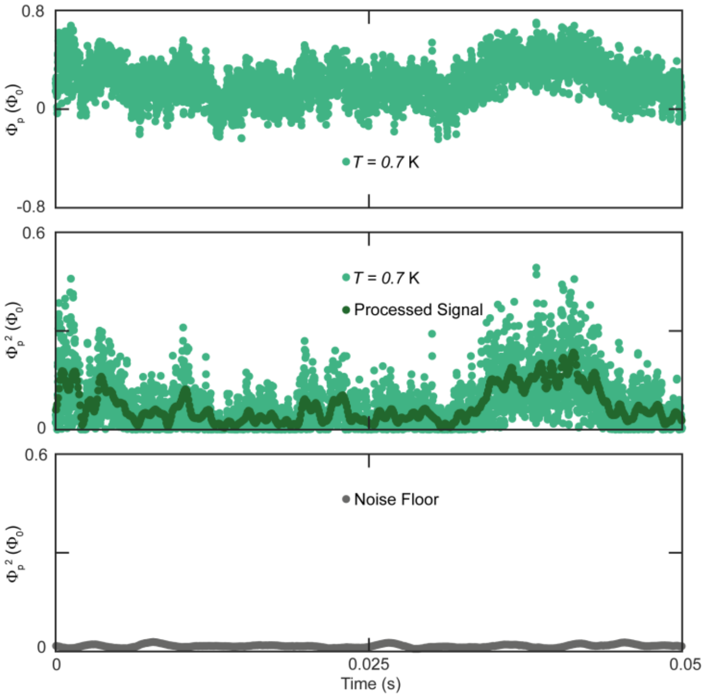

Fig. S7. Note that $\boldsymbol{\Phi_p^2}$ and energy are considered equivalent here due to their linear relationship as described in Eqn. S16. (Top) A typical flux signal $\boldsymbol{\Phi_p}$ measured at 700 mK. (Middle) The square of the flux signal $\boldsymbol{\Phi_p^2}$ is calculated and the signal is then averaged in an 80 μs window. The averaged signal is layered on top of $\boldsymbol{\Phi_p^2}$. (Bottom) The same routine is applied to the empty coil signal. The flux signal is considerably reduced in the empty coil data.

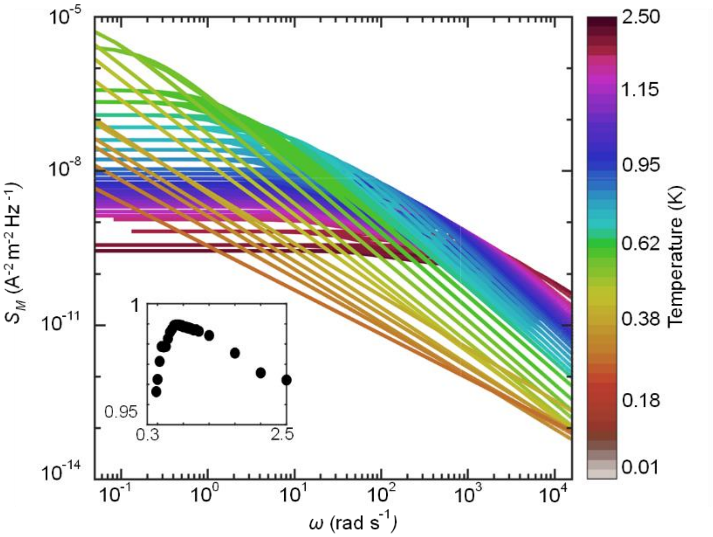

Fig. S8. Fitted magnetization noise power spectral density $\boldsymbol{S_M(\omega, T)}$ data versus $\boldsymbol{T}$. The noise is well described ($\boldsymbol{R^2 > 0.95}$) by monopole generation/recombination above $\mathbf{300\ mK}$. Below this temperature, fits are excluded.

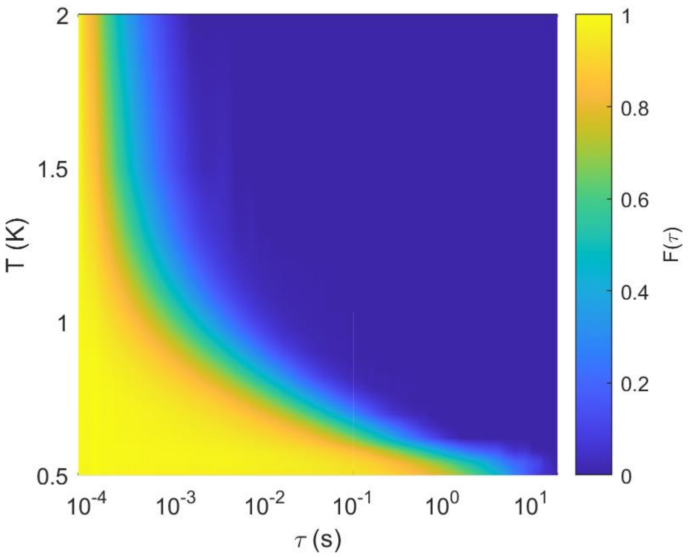

Fig. S9. Autocorrelation function data $\boldsymbol{F(\tau, T)}$ shown as a function of lag time $\boldsymbol{\tau}$ and temperature $\boldsymbol{T}$. $\boldsymbol{F(\tau, T)}$ shows the dramatically slowing dynamics of the monopoles with falling temperature. The increase in correlated monopole motion is evident as the monopole fluid enters the supercooled regime.

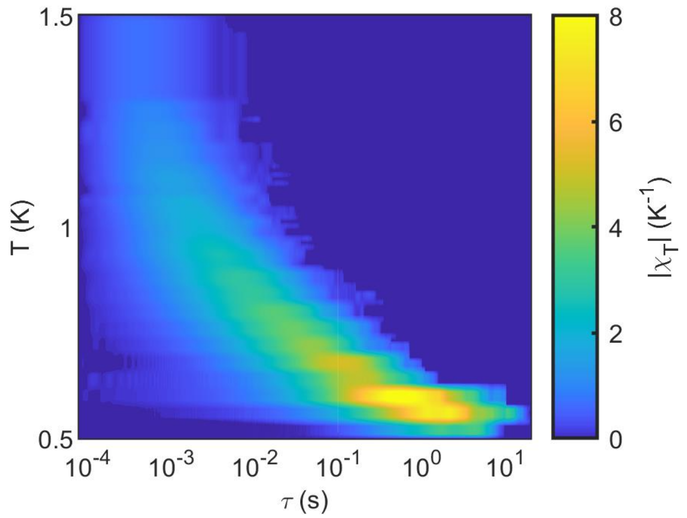

Fig. S10. Response function $\boldsymbol{\chi_T(\tau, T) = \partial F(\tau, T)/\partial T}$ data shown as a function of lag time $\boldsymbol{\tau}$ and temperature $\boldsymbol{T}$, directly calculated from the data in Fig. S9.

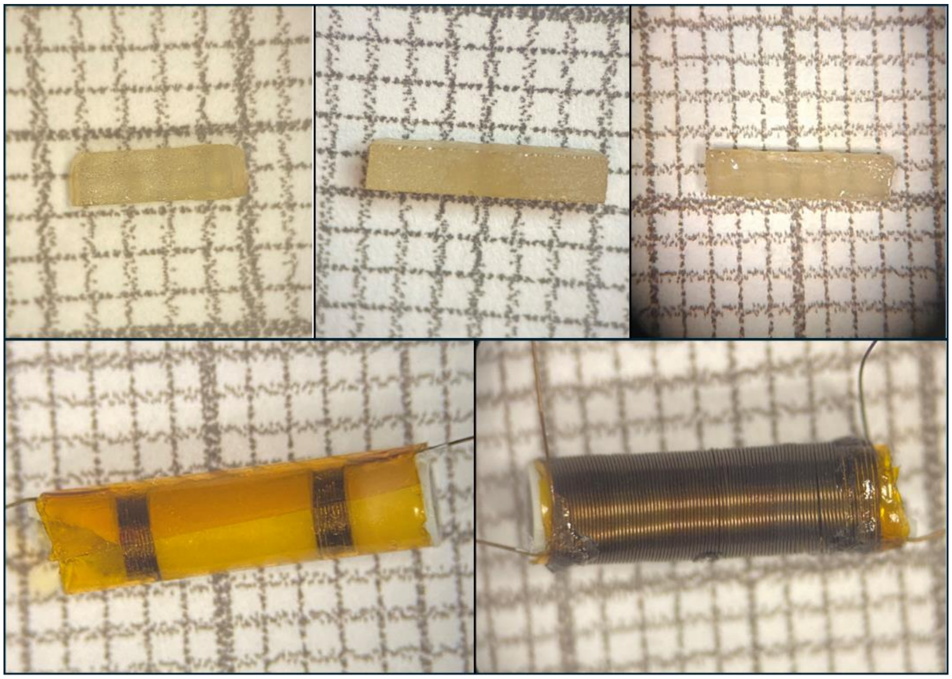

Fig. S11. Top Left: DTO Sample 1 with dimensions 0.74 mm x 1.48 mm x 4.97 mm. Top Middle: DTO Sample 2 with dimensions 0.98 mm x 1.31 mm x 6.23 mm. Top Right: DTO Sample 3 with dimensions 0.80 mm x 1.27 mm x 6.62 mm. Bottom Left: Pickup + Astatic coil assembly. The sample is inserted from the right into the pickup (rightmost) coil. Bottom Right: Drive coil wound around the pickup coil assembly. A layer of thin Kapton tape protects the inner wires while the drive coil is assembled.

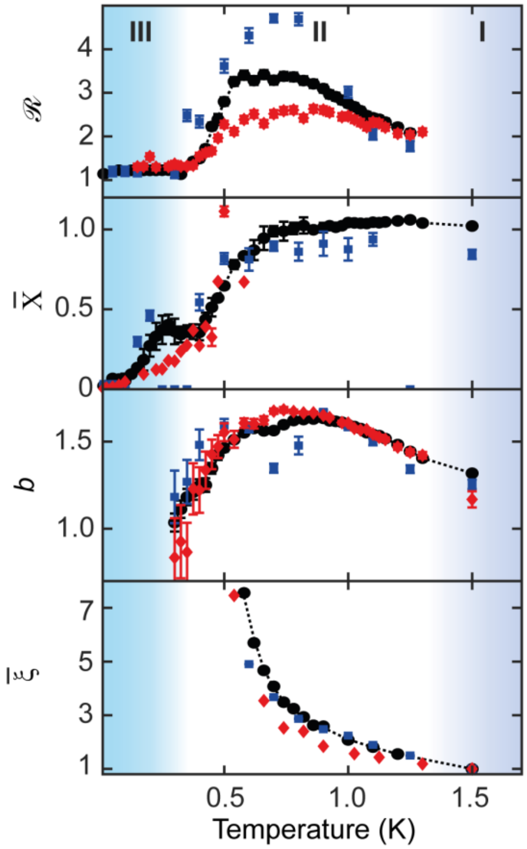

Fig. S12. Each sample studied in this work produced the same phenomenologies, demonstrating qualitative repeatability of the experiment. Sample 1 is shown as blue squares, Sample 2 is shown as black dots and Sample 3 is shown as red diamonds. Changes in magnitude of the noise can be attributed to geometric differences between samples.

Movie & Audio S1 (separate file). Top: The evolution of the flux noise $\phi_p(t, T)$ with falling temperature from $T = 2500$ mK to $T = 15$ mK. The flux noise signal, as it appears on screen, is converted to an audio signal and played over the video. Bottom: The simultaneous evolution of the monopole noise and monopole current burst energies at temperatures $15 \text{ mK} < T < 2500 \text{ mK}$.

SI References

52 M. Udagawa, L. Jaubert, *Spin Ice* (Springer, 2021).

53 D. Pomaranski *et al.,* Absence of Pauling's residual entropy in thermally equilibrated $Dy_2Ti_2O_7$. *Nat. Phys.* 9, 353-356 (2013).

54 C. Paulsen *et al.*, Far-from-equilibrium monopole dynamics in spin ice. *Nat. Phys.* 10, 135–139 (2014).

55 H. M. Revell *et al.,* Evidence of impurity and boundary effects on magnetic monopole dynamics in spin ice. *Nat. Phys*. 9, 34-37 (2013).

56 J. A. Quilliam, I. R. Yaraskavitch, H. A. Dabkowska, B. D. Gaulin, J. B. Kycia, Dynamics of the magnetic susceptibility deep in the Coulomb phase of the dipolar spin ice material $Ho_2Ti_2O_7$. *Phys. Rev. B* 83, 094424 (2011).

57 M. J. Jackson *et al.*, Dynamic behavior of magnetic avalanches in the spin-ice compound $Dy_2Ti_2O_7$. *Phys. Rev. B* 90, 064427 (2014).

58 C. Paulsen *et al.,* Nuclear spin assisted quantum tunnelling of magnetic monopoles in spin ice. *Nat Commun* 10, 1509 (2019).

59 K. Matsuhira *et al.,* Spin dynamics at very low temperatures in spin ice $Dy_2Ti_2O_7$. *J. Phys. Soc. Jpn* 80, 123711 (2011).

60 L. R. Yaraskavitch *et al.*, Spin dynamics in the frozen state of the dipolar spin ice $Dy_2Ti_2O_7$. *Phys. Rev. B* 85, 020410 (2012).

61 K. Matsuhira, Y. Hinatsu, T. Sakakibara, Novel dynamical magnetic properties in the spin ice compound $Dy_2Ti_2O_7$. *J. Phys.: Condens. Matter* 13, L737 (2001).

62 D. J. P. Morris *et al.,* Dirac Strings and Magnetic Monopoles in the Spin Ice $Dy_2Ti_2O_7$. *Science* 326, 411-414 (2009).

63 T. Stöter *et al.*, Extremely slow nonequilibrium monopole dynamics in classical spin ice. Phys. Rev. B 101, 224416 (2020).

64 J. Snyder *et al.*, Low-temperature spin freezing in the $Dy_2Ti_2O_7$ spin ice. *Phys. Rev. B* 69, 064414 (2004).

65 L. Bovo, J. A. Bloxsom, D. Prabhakaran, G. Aeppli, S. T. Bramwell, Brownian motion and quantum dynamics of magnetic monopoles in spin ice. *Nat. Commun* 4, 1535-1542 (2013).

66 J. Snyder, J. S. Slusky, R. J. Cava, P. Schiffer, How 'spin ice' freezes. *Nature* 413, 48-51 (2001).

67 J. Shi *et al.*, Dynamical magnetic properties of the spin ice crystal $Dy_2Ti_2O_7$. *J. Magn. Magn. Mater*. 310, 1322-1324 (2007).

68 R. A. Borzi *et al*. Intermediate magnetization state and competing orders in $Dy_2Ti_2O_7$ and $Ho_2Ti_2O_7$. *Nat. Commun* 7, 12592 (2016).

69 P. K. Yadav, C. Upadhyay, Quantum criticality in geometrically frustrated $Ho_2Ti_2O_7$ and $Dy_2Ti_2O_7$ spin ices. *J. Magn. Magn. Mater.* 482, 44-49 (2019).

70 H. Takatsu *et al.*, Universal dynamics of magnetic monopoles in two-dimensional Kagomé ice. *J. Phys. Soc. Jpn* 90, 123705 (2021).

71 A. B. Eyvazov *et al.,* Common glass-forming spin-liquid state in the pyrochlore magnets $Dy_2Ti_2O_7$ and $Ho_2Ti_2O_7$. *Phys. Rev. B* 98, 214430 (2018).

72 S. Giblin, S. T. Bramwell, P. C. W. Holdsworth, D. Prabhakaran, I. Terry, Creation and measurement of long-lived magnetic monopole currents in spin ice. *Nat. Phys*. 7 252-258 (2011).

73 M. Orendáč *et al.,* Magnetocaloric study of spin relaxation in dipolar spin ice. *Phys. Rev B* 75, 104425 (2007).

74 C. Donati, S. Franz, S. C. Glotzer, G. Parisi, Theory of non-linear susceptibility and correlation length in glasses and liquids. *J. Non-Cryst. Solids* 307-310, 215 (2002).

75 J. P. Hansen, I. R. Mc Donald, *Theory of Simple Liquids* (Elsevier, Amsterdam, 1986).

76 J. L. Lebowitz, J. K. Percus, L. Verlet, Ensemble Dependence of Fluctuations with Application to Machine Computations. *Phys. Rev.* 153, 250 (1967).

77 G. Tarjus, D. Kivelson, S. Mossa, C, Alba-Simionesco, Disentangling density and temperature effects in the viscous slowing down of glassforming liquids*. J. Chem. Phys* 120, 6135-6141 (2004).

78 L. Jaubert, P. C. W. Holdsworth, Signature of magnetic monopole and Dirac string
dynamics in spin ice. *Nat. Phys.* 5, 258–261 (2009).

79 K. Binder, A. P. Young, Spin. Glasses: Experimental facts, theoretical concepts, and open questions. *Rev. Mod. Phys*. 58, 901 (1986).

80 C. Crauste-Thibierge *et al*., Evidence of Growing Spatial Correlations at the Glass Transition from Nonlinear Response Experiments. *Phys. Rev. Lett*. 104, 165703 (2010).

81 Th. Bauer, P. Lunkenheimer, A. Loidl, Cooperativity and the Freezing of molecular Motion at the Glass Transition, *Phys. Rev. Lett*. 111, 225702 (2013).

82 S. Albert *et al*., Fifth-order susceptibility unveils growth of thermodynamic amorphous order in glass-formers. *Science* 352, 1308 (2016).

83 U. Tracht *et al*., Length Scale of Dynamic Heterogeneities at the Glass Transition Determined by Multidimensional Nuclear Magnetic Resonance. *Phys. Rev. Lett*. 81, 2727 (1998).

84 A. Smith, *et al.,* The demagnetizing field of a nonuniform rectangular prism. *J. Appl. Phys.* 107, 103910 (2010).

85 A. Ghasemi, A. Scheie, J. Kindervater, S. M. Koohpayeh, The pyrochlore Ho2Ti2O7: Synthesis, crystal growth, and stoichiometry. *J. Cryst. Growth* 500, 38 (2018).

86 C. Castelnovo, R. Moessner, S. L. Sondhi, Debye-Hückel theory for spin ice at low temperature. *Phys. Rev. B* 84, 144435 (2011).
